# Toll-like receptor-mediated innate immunity against herpesviridae infection: a current perspective on viral infection signaling pathways

**DOI:** 10.1186/s12985-020-01463-2

**Published:** 2020-12-09

**Authors:** Wenjin Zheng, Qing Xu, Yiyuan Zhang, E. Xiaofei, Wei Gao, Mogen Zhang, Weijie Zhai, Ronaldjit Singh Rajkumar, Zhijun Liu

**Affiliations:** 1 School of Basic Medical Sciences, Weifang Medical University, Weifang, 261053 China; 2 School of Anesthesiology, Weifang Medical University, Weifang, 261053 China; 3 Department of Microbiology and Physiological Systems, University of Massachusetts Medical School, Worcester, MA 01605 USA; 4 Key Lab for Immunology in Universities of Shandong Province, School of Basic Medical Sciences, Weifang Medical University, Weifang, 261053 China; 5 Department of Medical Microbiology, School of Basic Medical Sciences, Weifang Medical University, Weifang, 261053 China

**Keywords:** Herpesviridae, Toll-like receptor, Immune mechanism, Viral infection

## Abstract

**Background:**

In the past decades, researchers have demonstrated the critical role of Toll-like receptors (TLRs) in the innate immune system. They recognize viral components and trigger immune signal cascades to subsequently promote the activation of the immune system.

**Main body:**

Herpesviridae family members trigger TLRs to elicit cytokines in the process of infection to activate antiviral innate immune responses in host cells. This review aims to clarify the role of TLRs in the innate immunity defense against herpesviridae, and systematically describes the processes of TLR actions and herpesviridae recognition as well as the signal transduction pathways involved.

**Conclusions:**

Future studies of the interactions between TLRs and herpesviridae infections, especially the subsequent signaling pathways, will not only contribute to the planning of effective antiviral therapies but also provide new molecular targets for the development of antiviral drugs.

## Background

Toll-like receptors (TLRs) are a group of single, membrane-spanning, non-catalytic proteins in the immune system that are critical for recognizing structurally-conserved molecules derived from pathogenic microbes. To date, thirteen members have been identified in the TLR family. TLRs 1–10 are found in the human genome, and TLRs 11–13 occur in mice [1–6]. The structures of TLRs and other TLR-ligand complexes have been described [7–15]. Leucine-rich repeats have been described in the variable N-terminal extracellular part of TLRs, and have been shown to bind pathogen-associated molecular patterns, which are broadly shared by pathogens but not the host. This interaction allows the host to discriminate autologous from xenogenous substances [16].

TLRs are mainly expressed on the membranes of immune cells including macrophages, dendritic cells, T cells, and B cells [17–22]. Moreover, TLRs are also found in non-immune cells, such as endothelial and epithelial cells, adipocytes, and cardiomyocytes [23–27]. TLRs predominantly occur on the cell surface, while TLRs 3, 7, 8, and 9 are expressed inside cells [3]. These four TLRs are primarily involved in the identification of xenogenous nucleic acids from invaders. The cellular localizations and ligands of human TLRs 1–9 are listed in Table 1.

**Table 1:** Properties of toll-like receptors

TLRs	Localization	Ligands
TLR1/2 [33–35]	Cell surface	Triacylated lipopeptides
TLR2/6 [36–41]	Cell surface	Diacylated lipopeptides (*Mycoplasma*), Lipoteichoic acid (*Streptococcus*), Zymosan (*Saccharomyces cerevisiae*)
TLR2 [38, 42–48]	Cell surface	Peptidoglycan (Gram-positive bacteria), Lipoarabinomannan (Mycobacteria), Hemagglutinin (measles virus), phosphatidylinositol mannoside 6 (Mycobacteria), Glycosylphosphatidylinositol (*Trypanosoma*)
TLR3 [49–52]	Endosome	ssRNA virus (West Nile virus), dsRNA virus (Respiratory syncytial virus, murine cytomegalovirus)
TLR4 [43, 53–59]	Cell surface	Lipopolysaccharide (Gram-negative bacteria), Mannan-binding lectin (*Candida albicans*), glycoinositol- phospholipids (*Trypanosoma cruzi*), Envelope proteins (respiratory syncytial virus, mouse mammary tumor)
TLR5 [60, 61]	Cell surface	Flagellin (flagellated bacteria)
TLR7 [62, 63]	Endosome	ssRNA viruses (vesicular stomatitis virus, influenza virus)
TLR8 [64–66]	Endosome	ssRNA from RNA viruses
TLR9 [67–71]	Endosome	dsRNA viruses (herpes simplex virus, murine cytomegalovirus), CpG motifs from bacteria and viruses, Hemozoin (*Plasmodium*)

The herpesviridae family comprises a large group of enveloped DNA viruses characterized by latent infection in their hosts. Currently, eight family members are known to be associated with widespread human infection (Table 2). Upon detecting members of this family, TLRs recruit adaptor proteins, including myeloid differentiation factor 88 (MyD88), TIR-domain-containing adaptor-inducing interferon-β (TRIF), TIR-domain-containing adaptor protein (TIRAP), and TRIF-related adapter molecule (TRAM). This is followed by signal transmission to activate transcription factors including nuclear factor kappa B (NF-κB), activator protein-1 (AP-1), and interferon regulatory factors (IRF3/7). These factors enter the nucleus, stimulating transcription to promote pro-inflammatory cytokines and interferon (IFN) expression [5, 6, 28]. The inflammatory cascades defend against viruses while also injuring the host. Under physiological conditions, regulatory systems function in the host to inhibit excessive activation of the TLR signaling pathways to maintain homeostasis; these include Annexin A2, the ubiquitin ligase TRIAD3A, RP105, and acetylation of lysine residues [29–32]. Here, we clarify the mechanism underlying the human TLR-mediated innate immune response against herpesviridae in the activation and reactivation of virus infection.

**Table 2:** Properties of the herpesviridae family

Herpesviridae family members	Corresponding TLRs
Herpes simplex virus type 1 (HSV-1) [72–75]	TLR2, TLR3, TLR4, TLR9
HSV-2 [76, 77]	TLR2, TLR3, TLR4, TLR9
Varicella zoster virus [78, 79]	TLR2, TLR3, TLR9
Epstein-Barr virus [80–83]	TLR2, TLR3, TLR7, TLR9
Cytomegalovirus [84, 85]	TLR2, TLR3, TLR4, TLR5, TLR9
Human herpesvirus 6 (HHV-6) [86–89]	TLR4
HHV-7 [86]	TLR2, TLR4
Kaposi's sarcoma-associated herpesvirus [90–93]	TLR3, TLR4, TLR9

## Main text

### Herpes simplex virus

Herpes Simplex Virus (HSV) infection is a worldwide cause of severe medical conditions such as encephalitis, keratitis, and neonatal herpes [94, 95]. It has two serotypes, HSV-1 and HSV-2, which primarily infect individuals through epithelial cells. After initial infection, it forms a latent infection in ganglia and latency-associated transcripts are expressed [96]. HSV US3 protein inhibits TLR3 responses in cultured human monocytes [97]. Similarly, HSV immediate-early ICP0 protein suppresses the TLR2-mediated innate immune response and NF-κB signaling [98]. HSV downregulates TLR2 and TLR4 in a THP-1 monocyte cell line [99]. These findings reveal the evasion mechanism of HSV. When host immunity is weak, HSVs begin to reactive to establish infection.

Studies have revealed that TLR2, TLR3, TLR4, and TLR9 are capable of recognizing specific components of HSV such as glycoprotein B (gB), glycoprotein H (gH), glycoprotein K (gK), glycoprotein L (gL), and US2 protein in the activation and reactivation of HSV [100–105]. TLR signaling activates the transcription of immune response genes by inducing the secretion of intracellular protein signaling molecules such as interleukins (ILs) and interferons (IFNs) to protect the host. Furthermore, TLR2 and TLR9 have been shown to synergistically fuel innate immunity to defend against HSV-1 and -2, showing a protective effect [102, 106].

### Interactions of HSV with TLR2 and TLR4

Upon invasion of HSV-1 and -2, viral glycoproteins including gH and gL are recognized by TLR2 [107]. TLR2 is located on the dendritic cell surface and hetero-dimerizes with TLR6 or TLR1 to recognize viral glycoproteins [108]. Once HSV-2 has invaded the host, TLR4 recognizes the short-hairpin DNA from HSV on the cell surface [109]. Villalba et al. reported that TLR2 and TLR4 expression occurs as early as 1 h after HSV-1 infection and increase the levels of IRF3, IRF7, INF-β, and IL-6 [110]. The activation of TLR2 or TLR4 launches the MyD88-dependent signaling cascades and assembles macrophages and natural killer cells [109, 111]. MyD88 recruits IL-1 receptor-associated kinase 1 (IRAK1), then activates tumor necrosis factor receptor-associated factor (TRAF6) [112–115]. Subsequently, transforming growth factor-β-activated protein kinase-1-binding protein-2 (TAB2) and transforming growth factor-β-activated kinase-1 (TAK1) are recruited to stimulate the inhibitor of nuclear factor κB kinase (IKK) complex which comprises IκB kinase α (IKKα), IKKβ, and IKKγ (NEMO) [113, 116, 117]. IKKα serves as a stimulator of NF-κB in the IKK complex. In contrast, IKKβ phosphorylates and degrades the inhibitor of NF-κB (IκB) to release NF-κB [118, 119]. Alternatively, mitogen-activated protein kinases (MAPKs) are triggered by TAK1 to allow AP-1 into the nucleus [120–124]. NF-κB and AP-1 enable immune cells to secrete IL-15, TNF-α, and IFN to defend against HSV and counteract viral absorption. In addition, studies have demonstrated that the expression of chemokines, such as chemokine (C–C motif) ligands 7, 8, and 9, as well as chemokine (C-X-C motif) ligands 1, 2, 4, and 5, which play important roles in the innate immune response against HSV [125, 126]. Surprisingly, when activated via the TLR4-MyD88 axis, AP-1 upregulates TLR4 expression by feedback in genital epithelial cells to enhance immunity in humans [127]. A study has also shown that Sp1 has a significant effect as a major transcription factor involved in TLR2 promoter activity [107, 128].

Moreover, Kurt-Jones et al. demonstrated that neonates produce more pro-inflammatory cytokines than adults, which may explain the sepsis syndrome that is seen with HSV-1 and -2 [129]. This result is in accord with the finding that TLR2-deficient mice are more likely to survive HSV-1 than wild-type mice [105]. Besides the cytokine response, TLR2 signaling generates reactive oxygen species and induces oxidative stress, which cause damage in wild-type microglial cell cultures; but this does not occur in cells from TLR2-deficient mice. The consequences of oxidative stress are associated with reduced activation of the MAPK pathway [130]. These results suggest that the immune response mediated by TLR-2 can be not only beneficial but also detrimental to the host [105]. Surprisingly, TLR2 and TLR9 synergistically activate the innate antiviral response defense against HSV-1 and -2, showing a protective effect [106]. Compared to TLR2, TLR3 seems to have a protective effect [131, 132].

### Interactions between HSV and TLR3

**Fig. 1:**
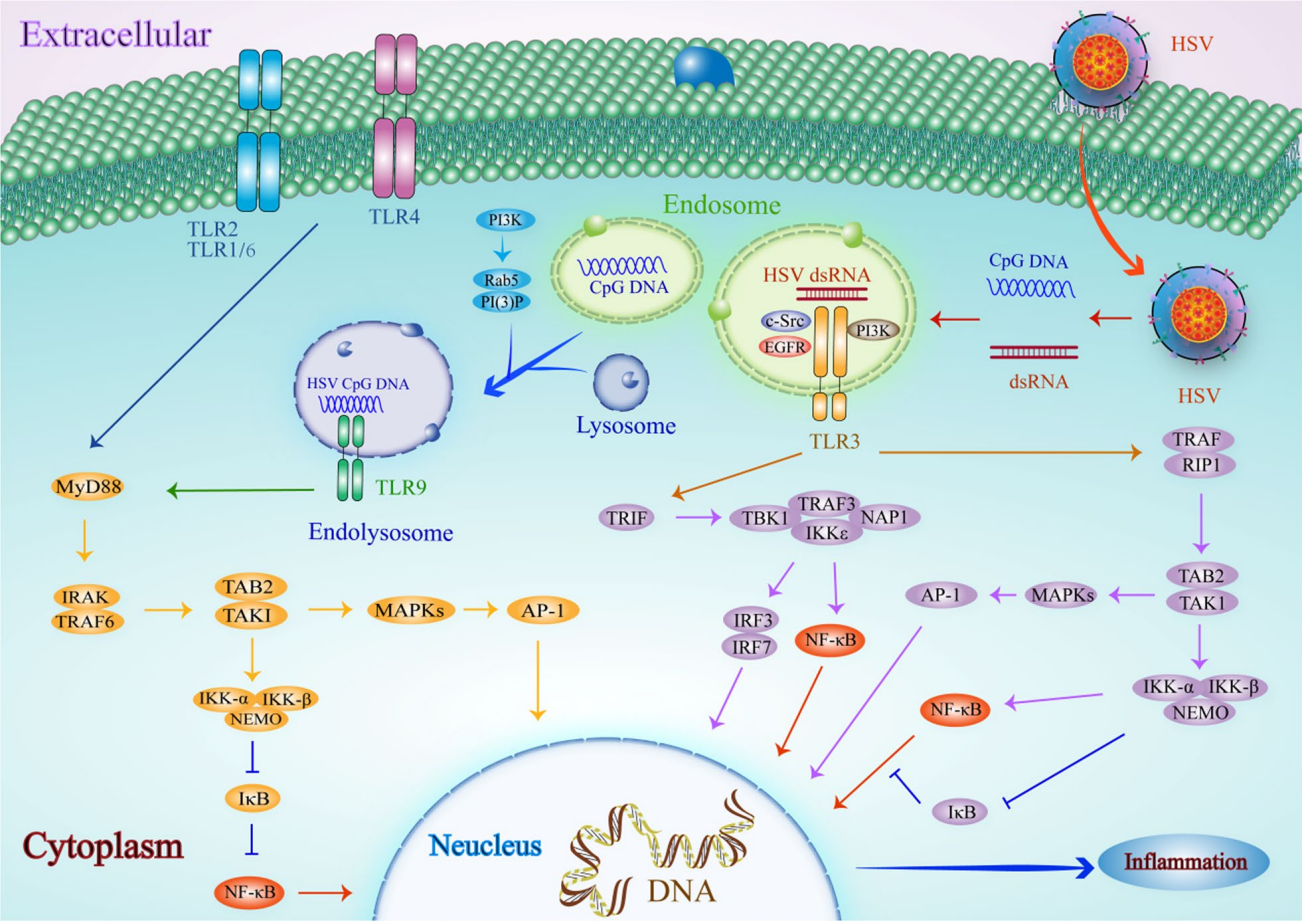
TLR-mediated signaling pathways in response to HSV**.** Upon HSV ligand stimulation, TLR2, TLR4, and TLR9 recruit the adaptor MyD88. Once recruited, MyD88 binds the protein complex composed of IRAK and TRAF6. TRAF6 results in the phosphorylation of TAK1, which then activates the IKK complex that results in the phosphorylation and degradation of IκB. The degradation of IκB allows NF-κB to translocate into the nucleus. Alternatively, TAK1 activates the MAPK pathway, triggering the activation of AP-1. Under HSV stimulation, TLR3 is localized and phosphorylated by tyrosine kinase c-Src, EGFR, and PI3K in the endosome. Moreover, TLR3 triggers TRIF to enable TBK1, IKKε, NAP1, and TRAF3 to generate a complex. Furthermore, this complex leads to the activation of IRF3/IRF7 and NF-κB. TLR3 recruits TRAF and RIP1 to phosphorylate TAB2 and TAK1. The complex formed by TAB2 and TAK1 activates AP-1 via the MAPK pathway and NF-κB via the IKK complex-IκB pathway. Together, NF-κB, IRF3/IRF7 and AP-1 induce the expression of inflammatory cytokines to protect the host by innate immunity

Upon identification of invasive HSV-1 and -2, the host cells form endosomes that spontaneously wrap up the virus. Unc-93 homolog B1 (UNC-93B) is a transmembrane protein localized on the endoplasmic reticulum (ER) that transfers TLR3, 7, 8, and 9 from the ER to the endosome [133–136]. Upon HSV-1 and -2 stimulation, TLR3 interacts with UNC-93B1 and shifts from the ER to endosome [134, 135]. In the endosome, TLR3 is phosphorylated by tyrosine kinase c-Src, epidermal growth factor receptor (EGFR), and phosphatidylinositol 3-kinase (PI3K) to form dimeric TLR3, which initiates a downstream signaling pathway. Although a mutual action between HSV RNA and TLR3 has not yet been demonstrated, it is likely that HSV-1 and -2 produce dsRNA that serves as a ligand for TLR3 [137–147]. The activation of TLR3 recruits TRIF and TRAF [148, 149]. TLR3 is the only member of the TLR family that can recruit TRIF and TRAF as the signal transduction factor, instead of MyD88. Upon TRIF recruitment, TANK-binding kinase-1 (TBK1), inhibitor of nuclear factor κB kinase ε (IKKε), NAK-associated protein 1 (NAP1), and TRAF3 constitute a signaling complex that leads to the activation of IRF3/IRF7 and NF-κB [150–157]. The activation of IRF3 and NF-κB induces the production of IFN-β, TNF-α, and IL-6 [158, 159]. Meanwhile, TRAF recruits the downstream protein receptor interacting protein 1 (RIP1), which subsequently recruits TAB2 and TAK1 to form a complex to trigger IKKα and IKKβ [160–162]. These two kinases with the IKK receptor protein IKKγ (NEMO) constitute the IKK complex [163]. IKKα activates downstream NF-κB, while IKKβ phosphorylates the inhibitor of NF-κB (IκB) leading to the degradation of IκB [119, 161]. The complex formed by TAB2 and TAK1 also activates AP-1 via MAPKs [164]. Subsequently, NF-κB, AP-1, and IRF3/IRF7 enter the nucleus and facilitate the release of IFN-β, TNF-α, and IL-6 to defend against HSV [165, 166] (Fig. 1).

### Interactions between HSV and TLR9

TLR9 is one of the crucial components in the defense against HSV-1 and -2. Similar to TLR3, TLR9 is stabilized by UNC-93B1 through preventing its degradation and transporting it from the ER to the endosome [136]. This redistribution of TLR9 is associated with cytosine-phosphate-guanine DNA (CpG DNA). Both HSV DNA and CpG oligonucleotides contain abundant CpG motifs [167, 168]. CpG DNA drives TLR9 to shift into early endosomes and CpG oligonucleotides access the endosome. Subsequently, the oligonucleotides assemble and form a secondary structure near the core CpG motif to activate TLR9 [3, 169, 170]. Guanosine triphosphatases (Rab GTPases) mediate the maturation of endosomes. Upon maturation, endosomes that contain CpG DNA combine with lysosomes. The hallmark of the maturation of endosomes involves the formation of endolysosomes [171–173]. On the endosomal membrane, Rab5 mediates class III phosphatidylinositol-3 kinase to produce phosphatidylinositol-3 phosphate that interacts with Rab5 to regulate and promote the maturation of early endosomes [174–176]. Furthermore, MyD88 activates IRAK1/4 to trigger the protein TRAF6. Subsequently, TRAF6 recruits and activates TAK1 (transforming growth factor-β-activated kinase 1) through the K-63-linked poly-ubiquitination of TAK1 and TRAF6 [177–179]. TAK1 initiates downstream cascades, including MAPKs and the NF-κB-inducing kinase (NIK)-IKK-IκB signaling pathway [180, 181]. In this pathway, NF-κB is isolated and inactivated in the cytoplasm primarily by IκB. The proteolysis of IκB is regulated by the activation of IKKs including IKKα, IKKβ, and IKKγ [182, 183]. Activated IKKβ leads to the phosphorylation and proteolysis of IκB [182]. NF-κB is unlocked and subsequently enters the nucleus. These processes induce the activation of transcription factors such as AP-l and NF-κB, directly facilitating the downstream gene expression of IL-10, IL-12, TNF-α, and IFN-β [180, 184–186] (Fig. 1).

## Varicella zoster virus

Varicella zoster virus (VZV) causes chicken-pox in the primary infection. In elderly or immunosuppressed patients, reactive VZV can cause herpes zoster after latency [187]. During the latency, VZV downregulates the surface expression of the NKG2D ligands of ULBP2 and ULBP3, which reduce the activation of natural killer cells in the presence of VZV [188].

Studies have reported that TLR2, TLR3, and TLR9 play crucial roles in the activation and reactivation of VZV [189–191]. TLR9 induces plasmacytoid dendritic cells (pDCs) to secrete IFN-α via the MyD88 signaling pathway involved in infection by VZV [191]. In addition, VZV triggers monocytes and macrophages to produce NF-κB via TLR2 and allows the secretion of the antiviral factor IL-6, but TLR2, TLR3, and TLR4 are not involved in the IFN-α production induced by VZV infection [189, 192]. Besides, studies have demonstrated that TLR3 is involved in the recognition of VZV [193]. There is no evidence that the expression of TLRs on non-immune cells react to infection with VZV. However, unlike other herpesviruses, the cytokine response to VZV is species-specific. VZV does not induce cytokines in mouse embryonic fibroblasts or in a mouse cell line, but it does trigger NF-κB in a human cell line expressing a mouse TLR2 construct [189].

## Epstein-Barr virus

### Interactions of EBV with TLR2

Epstein-Barr virus (EBV/HHV-4) is primarily transmitted via saliva. It proliferates in oropharyngeal epithelial cells, infects B lymphocytes, and enters the bloodstream to cause systemic infection. During the latency, the EBV lytic protein BGLF5 targets TLR9 mRNA for degradation in EBV-infected B cells, reducing the function of TLR9 [194]. Moreover, BGLF5 also targets TLR2 in infected cells [195]. In addition, a late lytic tegument protein, BPLF1, prevents TLR-mediated IFN production [196]. Besides, EBV-encoded miRNAs inhibit the TLR signaling pathway [197].

In the activation and reactivation of EBV, a membrane receptor expressed on the surface of B lymphocytes, TLR2 unites with TLR1 or TLR6 to form a hetero-dimer, which combines with lipoproteins or lipopeptides to serve as an active signaling complex. The TLR heterodimer (TLR2/TLRx) is the key to recognizing EBV. Eric Gaudreault et al. found that infectious and UV-inactivated EBV induce NF-κB activation and the secretion of primary monocyte chemotactic protein in a TLR2-dependent manner [198]. TLR2 activation initiates the MyD88-dependent signaling cascades. MyD88 recruits IRAKs, including IRAK1 and IRAK4, which stimulate TRAF6 and phosphorylate IKK, IκB, and NF-κB [199].

### Interactions of EBV with TLR3

When EBV penetrates a cell, it transcribes small non-coding RNAs called EBERs by using the host RNA polymerase III, and TLR3 is activated in the ER. EBERs induce inflammatory responses through TLR3 and neural precursor cells, resulting in high levels of cytokines such as TNF-α and IL-6. In addition to acting as an inflammatory mediator, NF-κB is capable of upregulating the expression of EBERs and LMP1 (EBV latent membrane protein 1), thereby triggering moderate inflammation [200, 201]. EBERs promote LMP1 transcription through NF-κB. Conversely, MP1 also stimulates NF-κB to increase the expression of EBERs. This positive regulatory loop becomes a necessary driving force for the inflammatory–carcinogenic transformation of EBV-infected epithelial cells.

### Interactions of EBV with TLR7

Furthermore, the EBV genome encodes two membrane proteins, LMP1 and LMP2, that act as natural signals of B-cell activation. LMP1 and LMP2 are required for the interaction of the ligand with the CD40 receptor and B-cell receptor. Martin et al. found that the TLR signaling pathway is a third pathway for activated B lymphocytes [202]. They reported that, after EBV infection of B lymphocytes, EBV gene expression transcribes ssRNA that stimulates TLR7 signaling, resulting in up-regulation of the TLR7 and MyD88 genes to activate IRF-5 and IRF-7 [203]. IRF-5 and NF-κB synergistically trigger cytokine promoters to induce the production of inflammatory cytokines. Moreover, they also provide a signal equivalent to the CD40 ligand to promote B cell activation and expansion in the initial phase of EBV infection. Therefore, it has been suggested that, in the early stage of infection, EBV stimulates TLR7 signaling to promote the initial stage of B cell activation and expansion. Subsequently, EBV induces negative-regulatory factors of the TLR7 pathway, which are necessary for the establishment of latency.

### Interactions of EBV with TLR9

In the primary infection, EBV initiates progressive lytic infection by expressing BZLF-1, which is the immediate-early lytic EBV gene and regulates the productive replication of EBV [204]. CpG oligodeoxynucleotide 2006 triggers innate immunity via the TLR9 of B cells to substantially inhibit BZLF-1 mRNA expression in acute EBV infection ex vivo and in Akata Burkitt lymphoma cells with latent EBV infection stimulated by anti-IgG. This reaction is mediated by IL-12 and IFN-γ [205]. When triggering TLR9, B cells infected with EBV ex vivo efficiently transform by reducing the initiation of lytic EBV infection, and thereby reinforcing the maintenance of EBV latency [206].

**Fig. 2:**
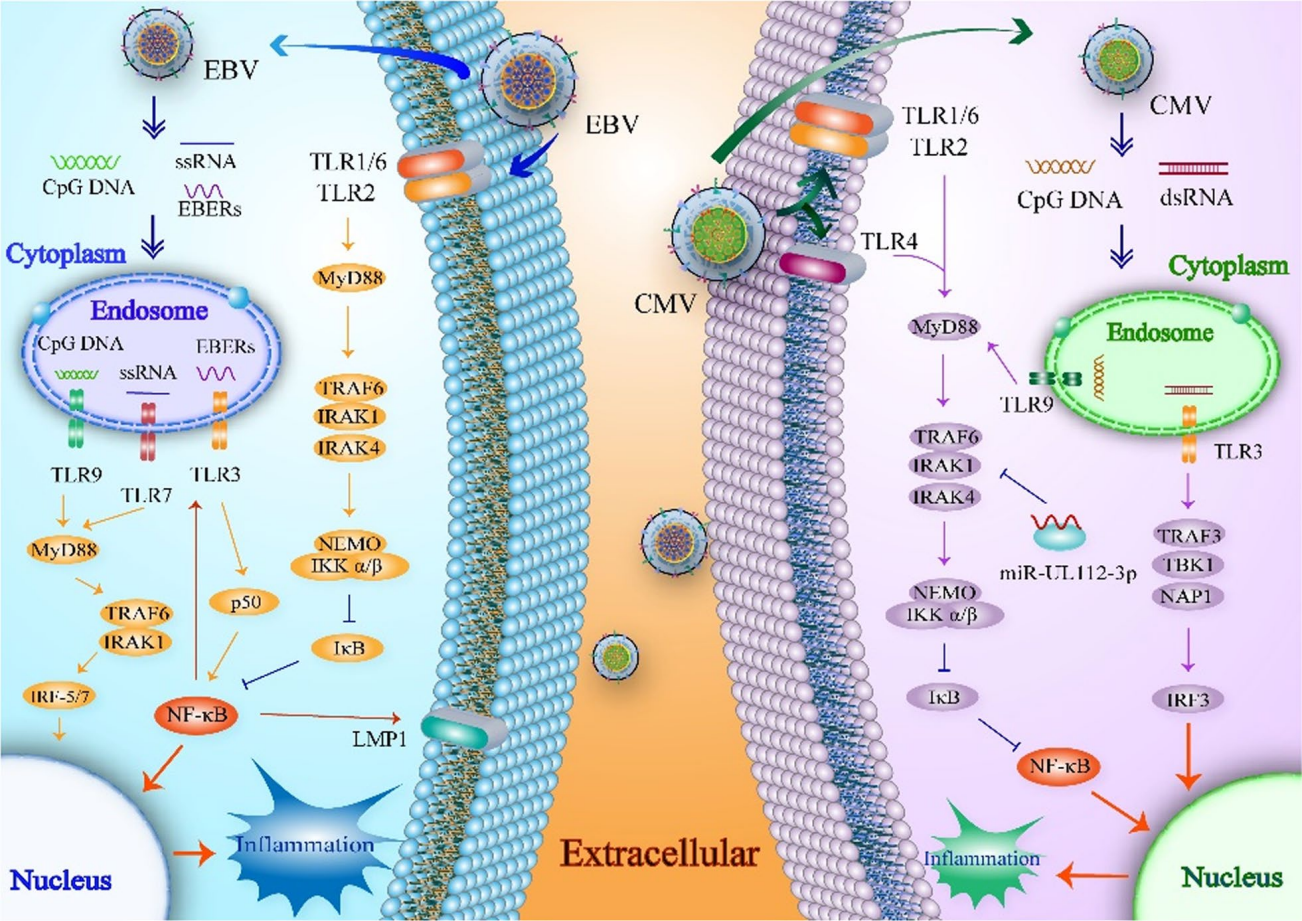
Mechanism of responses of TLRs to EBV and HCMV**.** EBV activates the MyD88 pathway or the MyD88-independent pathway via the viral envelope and products. Upon EBV stimulation, TLR2, TLR3, TLR7, and TLR9 inside and outside the cells induce NF-κB or IRF-5/7 by a series of protein kinases to produce cyto-inflammatory factors. MyD88 recruits TRAF6 and IRAKs to activate the IKK complex composed of IKKα, IKKβ, and NEMO. Besides acting as an inflammatory mediator, NF-κB also upregulates the expression of LMP1 to trigger moderate inflammation. Similarly, HCMV reacts with TLRs, including TLR2, TLR3, TLR4, and TLR9, through the viral envelope or products. MyD88-NF-κB is the main pathway. However, dsRNA from CMV also activates IRF-3 and TLR3 to promote the expression of inflammatory factors. Meanwhile, the CMV-encoded miR-UL112-3p inhibits activation of the TLR2/NF-κB pathway, as well as the expression of various cytokines

The newly-formed EBV DNA in virus-infected cells contains an unmethylated CpG dinucleotide sequence. When the newly-formed virion is subsequently released, this dinucleotide is considered to be the main trigger of TLR9 [207]. After TLR9 recognizes EBV DNA, IRAK-1 and TRAF6 are activated by phosphorylation, thereby eliciting the IKK complex, resulting in NF-κB expression [180]. Subsequently, NF-κB promotes the production of inflammatory cytokines such as TGF-β, IL-6, IL-1, IL-23, and IL-21 [207]. These cytokines induce Th17 cells to secrete IL-17A, causing the recruitment of neutrophils and macrophages to infected sites and triggering the secretion of various pro-inflammatory mediators by various cell types. Salloum et al. treated mouse peripheral blood mononuclear cells with EBV DNA in the presence or absence of the TLR9 inhibitor oligodeoxynucleotide 2088, and showed that TLR9 inhibitors significantly decrease IL-17A production and play a crucial role in promoting IL-17A secretion [208] (Fig. 2).

## Human cytomegalovirus

### Interactions of HCMV with TLR2

Human cytomegalovirus (HCMV) is an important cause of disease in the immunodeficient host and the most common intrauterine infection in humans [209]. Acquired during early life, HCMV persists in a latent state for the life of the individual. Inflammatory cytokines can cause an innate immune response in the host. Through different effector cells (such as antigen-presenting cells [APCs], natural killer [NK] cells and phagocytes), anti-inflammatory cytokines, and IFNs respond to act against HCMV infection. The early release of IFN-I and other pro-inflammatory cytokines limits the spread of infection by establishing an “antiviral state” that triggers an efficient adaptive immune response to achieve latency and persistence [210]. To achieve latency, the HCMV-encoded US7 and US8 proteins impair the activation of TLR3 and TLR4 [211]. Similarly, the HCMV-encoded US9 protein reduces stimulator of interferon genes (STING) signaling and the production of IFN [212]. In addition, the HCMV tegument protein UL82 inhibits STING-mediated signaling to evade the antiviral immune response [213].

Generally, researchers have shown that TLRs 2–5 and TLR9 play crucial roles in the immune response to the activation of HCMV [50, 67, 214–217]. TLR2 recognizes the viral envelope glycoproteins gB and gH. Together with TLR1 or TLR6, TLR2 activates the MyD88-dependent and downstream transcription factor NF-κB signaling pathway to induce a series of pro-inflammatory cytokines, chemokines, and adhesion molecules, such as IL-6 and IL-8 [218–220]. MyD88 recruits TRAF6 and IRAKs to activate IKKα and IKKβ, together with NEMO, to form the IKK complex. IKKα triggers downstream NF-κB, while IKKβ phosphorylates the NF-κB inhibitor IκB, leading to its degradation [160, 161, 163]. This process results in the production of inflammatory cytokines. For example, IFN-γ stimulates a variety of innate immune cells and immune effector cells to develop the adaptive immune response and exert an antiviral effect [221]. MicroRNAs are small non-coding RNAs that cooperate with viral proteins to regulate the expression of viral and/or host genes, and they are involved in the immune evasion of infected cells, as well as the latency and reactivation of HCMV [222]. CMV-encoded microRNAs have also been shown to downregulate TLR2 expression [217]. Using an *in-silico* method, this study postulated that HCMV microRNAs trigger the TLR innate immune pathway; specifically, TLR2 might be a target for HCMV miR-UL112-3p. Because miR-UL112-3p is expressed after virus entry, downregulation of TLR2 occurs in the late stage of lytic infection. Immunoblot analysis of miR-UL112-3p-transfected cells revealed that it induces the reduction of endogenous TLR2 expression. The microRNA-mediated downregulation of TLR2 affects innate signal transduction, significantly inhibiting the activation of the IRAK1 and NF-κB pathways located in the TLR2/NF-κB signaling axis of the upstream kinase, as well as the expression of various cytokines such as IL-1β, -6, and -8. TLR2 protein levels decrease in the late stage of HCMV infection, and this is associated with the accumulation of miR-UL112-3p in fibroblasts and mononuclear THP1 cells.

### Interactions of HCMV with TLR3, TLR4, and TLR5

TLR3 and TLR5 are also critical factors in the CMV infection pathway. TLR3 targets TRIF as a downstream adapter molecule instead of the adaptor protein MyD88 [149]. TLR3 activates the signaling complex assembled by TRIF. As a factor downstream to TRIF, TBK1 forms NAP1 and TRAF3 to elicit phosphorylation of the transcription factor IRF3, which produces inflammatory factors such as IFN-β [154–156, 221, 223]. CMV stimulates mast cells through the TLR3/TRIF signaling pathway to transmit effector functions. Subsequently, these cells release a large number of pro-inflammatory and antimicrobial mediators, many of which are stored in granules and released after degranulation, to enhance their protective properties and attract supplemental CD8 T cells to extravascular sites of viral replication [216]. During HCMV infection/reactivation, TLR5 plays an atypical role, probably because of the indirect effects of immunomodulation and immunostimulation on HCMV responses.

HCMV also promotes macrophage-mediated inflammatory responses through TLRs. HCMV infection stimulates cluster differentiation antigen 14 (CD14), TLR2, TLR4, and TLR5 on the surface to enhance the intracellular expression of the adaptor protein MyD88, and phosphorylation of IκB and NF-κB, thereby increasing the response of macrophages to viral components. The protein and mRNA levels of MyD88 are significantly elevated in macrophages. MyD88 combines with the cytoplasmic Toll/IL-1 region and triggers the phosphorylation of IRAK4, followed by the recruitment and phosphorylation of IRAK1, which then leads to the release of TNF-6 and transmission of the NF-κB signaling cascade [115, 224–228]. These mechanisms promote ligand-induced pro-inflammatory cytokine mRNA expression and the production of TNF-α, IL-6, and IL-8 proteins.

### Interactions of HCMV with TLR9

TLR9, a pattern recognition receptor for HCMV in natural IFN-producing cells and DCs, recognizes unmethylated CpG motifs in viral DNA to initiate the transduction of intracellular signals by the adapter molecule MyD88, ultimately leading to the activation and transcription of NF-κB. Therefore, phosphorylated NF-κB encodes pro-inflammatory cytokines and chemokines, such as IFN-α and IL-12, to promote NK cells that recognize MCMV-infected cells by activating the receptor Ly49H [229, 230]. Ly49H interacts with the MCMV-encoded protein m157 on the surface of infected cells, resulting in elimination of the virus by NK cells [67] (Fig. 2).

## Human herpesvirus-6 and -7

### Interaction between HHV-6 and TLRs

HHV-6 causes the exanthema subtype; it preferentially infects functional immune cells and elicits various immunobiological changes [231–234]. Murakami et al. pointed out that HHV-6 infection significantly effects TLR4-induced cytokine levels [235]. This report revealed that TLR4 and the adaptor molecule MyD88 are significantly increased in HHV-6-infected cells. On the contrary, the phosphorylation levels of TAK-1, IKKα/β, and IκBα are reduced and affect the expression of NF-κB [236]. Therefore, upon stimulation of the TLR4 ligand, the ability of HHV-6-infected DCs to produce IL-10 and IL-8 is significantly impaired. This indicates that, in HHV-6-infected DCs, the disruption of TLR4 signaling is caused by a block in the downstream signaling pathway.

### Interaction between HHV-7 and TLRs

HHV-6 and -7 participate in the pathogenesis of pityriasis rosea through TLRs. In HHV-7-positive cases, the expression levels of TLR2 and TLR4 are notably increased, while TLR9 and the HHV-7 viral load are positively correlated [237]. Interestingly, there is an interaction between HHV-6 and HHV-7: HHV-6 can be reactivated by HHV-7 infection [238].

## Kaposi's sarcoma-associated herpesvirus

Kaposi's sarcoma-associated herpesvirus (KSHV), also named human herpesvirus-8, is well correlated with several forms of cancer such as Kaposi's sarcoma, primary effusion lymphoma, and multicentric Castleman’s disease [239]. Like other herpesviruses, KSHV also causes latency in the host. During the latency, KSHV viral interferon regulatory factors (vIRFs) inhibit TLR3-mediated IFN induction [240]. Moreover, the replication and transcription activator (RTA) protein from KSHV triggers proteasomal degradation of the TLR3 adaptor protein TRIF, which blocks the subsequent pathway [241]. RTA also prevents TLR4 signaling via the degradation of MyD88 [242]. West et al. first reported that KSHV upregulates the TLR3 pathway during infection to induce TLR3-specific cytokines and chemokines such as IFN-1β and CXCL10 (IP-10) [243]. Furthermore, researchers have determined that TLR9 is the major receptor for KSHV. Once pDCs are infected, KSHV upregulates TLR9, CD83, and CD86, causing pDCs to produce IFN-α [244].

In addition, TLR4 plays an essential role in innate immunity to KSHV. KSHV microRNA clusters (particularly miRNA-K1, -K3, and -K11) trigger TLR4 with its co-receptors, CD14 and myeloid differentiation protein 2, to activate the MyD88-NF-κB pathway and produce IL-1β, IL-6, and IL-18 [245]. In addition, Lagos et al. found that KSHV suppression of TLR4 expression is the mechanism of immune escape during KSHV infection in endothelial cells [246]. Moreover, KSHV inhibits the TLR2 signaling pathway after infection in macrophages. In addition, the replication of KSHV and the transcriptional activator RTA/ORF50 block the TLR2 and TLR4 signaling pathways via reducing the expression of functional proteins. Moreover, KSHV-encoded microRNAs reduce the inflammatory factor expression by modulating two components of the TLR/IL-1R pathway, IRAK1 and MyD88 [247]. Thus, KSHV uses two mechanisms to avoid attack by the host immune system, leading to repeated infection in the host [248].

## Conclusions

To date, studies have shed light on the interactions between TLRs and herpesviridae infections, especially the subsequent signaling pathways. Research continues to reveal new insights into TLR pathways and their roles in host defense responses, especially in innate immunity [249–251]. However, the detailed mechanisms of mutual action between HSV RNA and TLR3 remain unclear [138–140, 148]. Moreover, understanding the mechanisms of activation and regulation in detail will help in the design of efficient vaccines and therapeutics based on modulating the TLRs more precisely. In this context, the use of TLR antagonists and regulators such as MPL, topical SMIP-7.7, Annexin A2, ubiquitin ligase TRIAD3A, pathogenesis-related protein from *Oenanthe javanica*, and RP105 might have broader applications [29, 31, 32, 252–254]. Although computer-assisted screening of TLR regulators is plausible, the rational design of selective TLR modulators still faces enormous challenges and studies are few. Furthermore, there are some new developments in anti-viral targeting of the host factors involved in TLR signaling. BX795, an inhibitor TBK1, potently suppresses multiple strains of HSV-1, including an ACV-resistant HSV-1 strain. BX795 targets Akt and blocks viral protein synthesis by reducing Akt phosphorylation in infected cells, but a more precise antiviral mechanism requires further investigation [255]. Therefore, clarifying the interaction between each TLR and the associated virus is critical for controlling the development of the diseases caused by the herpesviruses.

## Data Availability

Not applicable.

## Abbreviations

TLRs: Toll-Like receptors
HSV-1,-2: Herpes simplex virus type 1 and 2
VZV: Varicella zoster virus
EBV: Epstein-Barr virus
CMV: Cytomegalovirus
HHV-6, -7: Human herpesvirus 6 and 7
KSHV: Kaposi's sarcoma-associated herpesvirus
PRRs: Pathogen recognition receptors
MyD88: Myeloid differentiation 88
TRIF: Toll/IL-1R(TIR)-domain-containing adaptor protein
TIRAP: TIR-domain-containing adaptor protein
TRAM: TRIF-related adaptor molecule
CpG: Cytosine-phosphate-guanine
PI3K: Phosphatidylinositol 3-kinase
TRAF: Tumor-necrosis-factor-receptor-associated factor
IRAK1: Interleukin-1 receptor-associated kinase 1
MAPKs: Mitogen-activated protein kinases
Akt (PκB): Protein kinase B
JNK: Jun N-terminal kinase
NAP1: NAK-associated protein 1
TBK1: Tumor-necrosis-factor receptor-associated factor (TRAF) family-member-associated NF-κB activator (TANK) binding kinase 1
TAK-1: Transforming growth factor-β (TGF-β)-activated kinase 1-related kinase
IKK: Inhibitor of NF-κB kinase
NF-κB: Nuclear factor-κB
TNF: Tumor-necrosis factor
IRF: Interferon regulatory factor
IFN: Interferon, CD14: cluster differentiation antigen 14
LMP1: EBV latent membrane protein 1
NEMO: IKK receptor protein IKKγ
pDCs: PlaSmacytoid dendritic cells
IL: Interleukin
PMC: Peripheral blood mononuclear cells
RIP1: Receptor interacting protein 1
LRRs: Leucine-rich repeats
UNC-93B: Unc-93 homolog B1
AP-1: Activator protein-1
APC: Antigen-presenting cells
NK: Natural killer
dsRNA: Double-stranded RNA
ssRNA: Single-stranded RNA
EGFR: Epidermal growth factor receptor
ERK: Extracellular receptor kinase
STING: Stimulator of interferon genes

## References

[1] Boehme KW, Compton T (2004). Innate sensing of viruses by toll-like receptors. J Virol.

[2] Mahla RS, Reddy MC, Prasad DV, Kumar H (2013). Sweeten PAMPs: role of sugar complexed PAMPs in innate immunity and vaccine biology. Front Immunol.

[3] Akira S, Uematsu S, Takeuchi O (2006). Pathogen recognition and innate immunity. Cell.

[4] Celhar T, Magalhaes R, Fairhurst AM (2012). TLR7 and TLR9 in SLE: when sensing self goes wrong. Immunol Res.

[5] Takeuchi O, Akira S (2010). Pattern recognition receptors and inflammation. Cell.

[6] Kawai T, Akira S (2010). The role of pattern-recognition receptors in innate immunity: update on toll-like receptors. Nat Immunol.

[7] Song WS, Jeon YJ, Namgung B, Hong M, Yoon SI (2017). A conserved TLR5 binding and activation hot spot on flagellin. Sci Rep.

[8] Gosu V, Son S, Shin D, Song KD (2019). Insights into the dynamic nature of the dsRNA-bound TLR3 complex. Sci Rep.

[9] Yoon SI, Kurnasov O, Natarajan V, Hong M, Gudkov AV, Osterman AL, Wilson IA (2012). Structural basis of TLR5-flagellin recognition and signaling. Science.

[10] Collins B, Wilson IA (2014). Crystal structure of the C-terminal domain of mouse TLR9. Proteins.

[11] Ohto U, Tanji H, Shimizu T (2014). Structure and function of toll-like receptor 8. Microbes Infect.

[12] Ohto U, Shibata T, Tanji H, Ishida H, Krayukhina E, Uchiyama S, Miyake K, Shimizu T (2015). Structural basis of CpG and inhibitory DNA recognition by Toll-like receptor 9. Nature.

[13] Maeda K, Akira S (2016). TLR7 structure: cut in Z-Loop. Immunity.

[14] Zhang Z, Ohto U, Shibata T, Krayukhina E, Taoka M, Yamauchi Y, Tanji H, Isobe T, Uchiyama S, Miyake K, Shimizu T (2016). Structural analysis reveals that toll-like receptor 7 is a dual receptor for guanosine and single-stranded RNA. Immunity.

[15] Su L, Wang Y, Wang J, Mifune Y, Morin MD, Jones BT, Moresco EMY, Boger DL, Beutler B, Zhang H (2019). Structural basis of TLR2/TLR1 activation by the synthetic agonist diprovocim. J Med Chem.

[16] Hallman M, Ramet M, Ezekowitz RA (2001). Toll-like receptors as sensors of pathogens. Pediatr Res.

[17] Jang AR, Choi JH, Shin SJ, Park JH (2018). Mycobacterium tuberculosis ESAT6 induces IFN-beta gene expression in Macrophages via TLRs-mediated signaling. Cytokine.

[18] Kugelberg E (2014). Dendritic cells: TLR agonists trigger rapid metabolic changes. Nat Rev Immunol.

[19] Zahm CD, Colluru VT, McIlwain SJ, Ong IM, McNeel DG (2018). TLR stimulation during T-cell activation lowers PD-1 expression on CD8(+) T Cells. Cancer Immunol Res.

[20] Flaherty S, Reynolds JM (2016). TLR function in murine CD4(+) T lymphocytes and their role in inflammation. Methods Mol Biol.

[21] Hua Z, Hou B (2013). TLR signaling in B-cell development and activation. Cell Mol Immunol.

[22] Naradikian MS, Myles A, Beiting DP, Roberts KJ, Dawson L, Herati RS, Bengsch B, Linderman SL, Stelekati E, Spolski R (2016). Cutting edge: IL-4, IL-21, and IFN-gamma interact To govern T-bet and CD11c expression in TLR-activated B cells. J Immunol.

[23] Akira S, Takeda K, Kaisho T (2001). Toll-like receptors: critical proteins linking innate and acquired immunity. Nat Immunol.

[24] Menden H, Xia S, Mabry SM, Noel-MacDonnell J, Rajasingh J, Ye SQ, Sampath V (2019). Histone deacetylase 6 regulates endothelial MyD88-dependent canonical TLR signaling, lung inflammation, and alveolar remodeling in the developing lung. Am J Physiol Lung Cell Mol Physiol.

[25] Thomalla M, Schmid A, Neumann E, Pfefferle PI, Muller-Ladner U, Schaffler A, Karrasch T (2019). Evidence of an anti-inflammatory toll-like receptor 9 (TLR 9) pathway in adipocytes. J Endocrinol.

[26] Yang Y, Sun Y, Xu J, Bao K, Luo M, Liu X, Wang Y (2018). Epithelial cells attenuate toll-like receptor-mediated inflammatory responses in monocyte-derived macrophage-like cells to mycobacterium tuberculosis by modulating the PI3K/Akt/mTOR signaling pathway. Mediators Inflamm.

[27] Liu ZW, Zhu HT, Chen KL, Qiu C, Tang KF, Niu XL (2013). Selenium attenuates high glucose-induced ROS/TLR-4 involved apoptosis of rat cardiomyocyte. Biol Trace Elem Res.

[28] Kawai T, Akira S (2007). TLR signaling. Semin Immunol.

[29] Zhang S, Yu M, Guo Q, Li R, Li G, Tan S, Li X, Wei Y, Wu M (2015). Annexin A2 binds to endosomes and negatively regulates TLR4-triggered inflammatory responses via the TRAM-TRIF pathway. Sci Rep.

[30] Hu X, Yu Y, Eugene Chin Y, Xia Q (2013). The role of acetylation in TLR4-mediated innate immune responses. Immunol Cell Biol.

[31] Divanovic S, Trompette A, Atabani SF, Madan R, Golenbock DT, Visintin A, Finberg RW, Tarakhovsky A, Vogel SN, Belkaid Y (2005). Inhibition of TLR-4/MD-2 signaling by RP105/MD-1. J Endotoxin Res.

[32] Chuang TH, Ulevitch RJ (2004). Triad3A, an E3 ubiquitin-protein ligase regulating Toll-like receptors. Nat Immunol.

[33] Nakata T, Yasuda M, Fujita M, Kataoka H, Kiura K, Sano H, Shibata K (2006). CD14 directly binds to triacylated lipopeptides and facilitates recognition of the lipopeptides by the receptor complex of Toll-like receptors 2 and 1 without binding to the complex. Cell Microbiol.

[34] Ranoa DR, Kelley SL, Tapping RI (2013). Human lipopolysaccharide-binding protein (LBP) and CD14 independently deliver triacylated lipoproteins to Toll-like receptor 1 (TLR1) and TLR2 and enhance formation of the ternary signaling complex. J Biol Chem.

[35] Turner ML, Cronin JG, Healey GD, Sheldon IM (2014). Epithelial and stromal cells of bovine endometrium have roles in innate immunity and initiate inflammatory responses to bacterial lipopeptides in vitro via Toll-like receptors TLR2, TLR1, and TLR6. Endocrinology.

[36] Schroder NW, Morath S, Alexander C, Hamann L, Hartung T, Zahringer U, Gobel UB, Weber JR, Schumann RR (2003). Lipoteichoic acid (LTA) of Streptococcus pneumoniae and Staphylococcus aureus activates immune cells via Toll-like receptor (TLR)-2, lipopolysaccharide-binding protein (LBP), and CD14, whereas TLR-4 and MD-2 are not involved. J Biol Chem.

[37] Into T, Kiura K, Yasuda M, Kataoka H, Inoue N, Hasebe A, Takeda K, Akira S, Shibata K (2004). Stimulation of human Toll-like receptor (TLR) 2 and TLR6 with membrane lipoproteins of Mycoplasma fermentans induces apoptotic cell death after NF-kappa B activation. Cell Microbiol.

[38] Roeder A, Kirschning CJ, Rupec RA, Schaller M, Weindl G, Korting HC (2004). Toll-like receptors as key mediators in innate antifungal immunity. Med Mycol.

[39] Mayer ML, Phillips CM, Townsend RA, Halperin SA, Lee SF (2009). Differential activation of dendritic cells by Toll-like receptor agonists isolated from the Gram-positive vaccine vector Streptococcus gordonii. Scand J Immunol.

[40] Hong SW, Baik JE, Kang SS, Yun CH, Seo DG, Han SH (2014). Lipoteichoic acid of Streptococcus mutans interacts with Toll-like receptor 2 through the lipid moiety for induction of inflammatory mediators in murine macrophages. Mol Immunol.

[41] Taghavi M, Mortaz E, Khosravi A, Vahedi G, Folkerts G, Varahram M, Kazempour-Dizaji M, Garssen J, Adcock IM (2018). Zymosan attenuates melanoma growth progression, increases splenocyte proliferation and induces TLR-2/4 and TNF-alpha expression in mice. J Inflamm (Lond).

[42] Schwandner R, Dziarski R, Wesche H, Rothe M, Kirschning CJ (1999). Peptidoglycan- and lipoteichoic acid-induced cell activation is mediated by toll-like receptor 2. J Biol Chem.

[43] Takeuchi O, Hoshino K, Kawai T, Sanjo H, Takada H, Ogawa T, Takeda K, Akira S (1999). Differential roles of TLR2 and TLR4 in recognition of gram-negative and gram-positive bacterial cell wall components. Immunity.

[44] Talreja J, Kabir MH (2004). M BF, Stechschulte DJ, Dileepan KN: Histamine induces Toll-like receptor 2 and 4 expression in endothelial cells and enhances sensitivity to Gram-positive and Gram-negative bacterial cell wall components. Immunology.

[45] Castillo C, Munoz L, Carrillo I, Liempi A, Medina L, Galanti N, Maya JD, Kemmerling U (2017). Toll-like receptor-2 mediates local innate immune response against Trypanosoma cruzi in ex vivo infected human placental chorionic villi explants. Placenta.

[46] Shukla S, Richardson ET, Drage MG, Boom WH, Harding CV: Mycobacterium tuberculosis lipoprotein and lipoglycan binding to toll-like receptor 2 correlates with agonist activity and functional outcomes**.** Infect Immun 2018, **86**. 10.1128/IAI.00450-18 PMC6204744 30037791

[47] Bieback K, Lien E, Klagge IM, Avota E, Schneider-Schaulies J, Duprex WP, Wagner H, Kirschning CJ, Ter Meulen V, Schneider-Schaulies S (2002). Hemagglutinin protein of wild-type measles virus activates toll-like receptor 2 signaling. J Virol.

[48] Gravina HD, Antonelli L, Gazzinelli RT, Ropert C (2013). Differential use of TLR2 and TLR9 in the regulation of immune responses during the infection with Trypanosoma cruzi. PLoS ONE.

[49] Wang T, Town T, Alexopoulou L, Anderson JF, Fikrig E, Flavell RA (2004). Toll-like receptor 3 mediates West Nile virus entry into the brain causing lethal encephalitis. Nat Med.

[50] Szomolanyi-Tsuda E, Liang X, Welsh RM, Kurt-Jones EA, Finberg RW (2006). Role for TLR2 in NK cell-mediated control of murine cytomegalovirus in vivo. J Virol.

[51] Satkunanathan S, Kumar N, Bajorek M, Purbhoo MA, Culley FJ (2014). Respiratory syncytial virus infection, TLR3 ligands, and proinflammatory cytokines induce CD161 ligand LLT1 expression on the respiratory epithelium. J Virol.

[52] Verma R, Bharti K (2017). Toll like receptor 3 and viral infections of nervous system. J Neurol Sci.

[53] Oliveira AC, Peixoto JR, de Arruda LB, Campos MA, Gazzinelli RT, Golenbock DT, Akira S, Previato JO, Mendonca-Previato L, Nobrega A, Bellio M (2004). Expression of functional TLR4 confers proinflammatory responsiveness to Trypanosoma cruzi glycoinositolphospholipids and higher resistance to infection with T. cruzi. J Immunol.

[54] Medeiros MM, Peixoto JR, Oliveira AC, Cardilo-Reis L, Koatz VL, Van Kaer L, Previato JO, Mendonca-Previato L, Nobrega A, Bellio M (2007). Toll-like receptor 4 (TLR4)-dependent proinflammatory and immunomodulatory properties of the glycoinositolphospholipid (GIPL) from Trypanosoma cruzi. J Leukoc Biol.

[55] Wang M, Wang F, Yang J, Zhao D, Wang H, Shao F, Wang W, Sun R, Ling M, Zhai J, Song S (2013). Mannan-binding lectin inhibits Candida albicans-induced cellular responses in PMA-activated THP-1 cells through Toll-like receptor 2 and Toll-like receptor 4. PLoS ONE.

[56] Plociennikowska A, Hromada-Judycka A, Borzecka K, Kwiatkowska K (2015). Co-operation of TLR4 and raft proteins in LPS-induced pro-inflammatory signaling. Cell Mol Life Sci.

[57] Jang JC, Li J, Gambini L, Batugedara HM, Sati S, Lazar MA, Fan L, Pellecchia M, Nair MG (2017). Human resistin protects against endotoxic shock by blocking LPS-TLR4 interaction. Proc Natl Acad Sci U S A.

[58] Ryu JK, Kim SJ, Rah SH, Kang JI, Jung HE, Lee D, Lee HK, Lee JO, Park BS, Yoon TY, Kim HM (2017). Reconstruction of LPS transfer cascade reveals structural determinants within LBP, CD14, and TLR4-MD2 for efficient LPS recognition and transfer. Immunity.

[59] Yuan X, Hu T, He H, Qiu H, Wu X, Chen J, Wang M, Chen C, Huang S (2018). Respiratory syncytial virus prolifically infects N2a neuronal cells, leading to TLR4 and nucleolin protein modulations and RSV F protein co-localization with TLR4 and nucleolin. J Biomed Sci.

[60] McHeik S, Al-Akl NS, Abdelnoor AM (2018). The effect of denatured flagellin on toll-like receptor-5 (TLR-5) in mice. Endocr Metab Immune Disord Drug Targets.

[61] Ahmed M, Mitchell LM, Puckett S, Brzoza-Lewis KL, Lyles DS, Hiltbold EM (2009). Vesicular stomatitis virus M protein mutant stimulates maturation of Toll-like receptor 7 (TLR7)-positive dendritic cells through TLR-dependent and -independent mechanisms. J Virol.

[62] To EE, Broughton BR, Hendricks KS, Vlahos R, Selemidis S (2014). Influenza A virus and TLR7 activation potentiate NOX2 oxidase-dependent ROS production in macrophages. Free Radic Res.

[63] Tian J, Jiao X, Wang X, Geng J, Wang R, Liu N, Gao X, Griffin N, Shan F (2018). Novel effect of methionine enkephalin against influenza A virus infection through inhibiting TLR7-MyD88-TRAF6-NF-kappaB p65 signaling pathway. Int Immunopharmacol.

[64] Heil F, Hemmi H, Hochrein H, Ampenberger F, Kirschning C, Akira S, Lipford G, Wagner H, Bauer S (2004). Species-specific recognition of single-stranded RNA via toll-like receptor 7 and 8. Science.

[65] Han X, Li X, Yue SC, Anandaiah A, Hashem F, Reinach PS, Koziel H, Tachado SD (2012). Epigenetic regulation of tumor necrosis factor alpha (TNFalpha) release in human macrophages by HIV-1 single-stranded RNA (ssRNA) is dependent on TLR8 signaling. J Biol Chem.

[66] Bernard MA, Han X, Inderbitzin S, Agbim I, Zhao H, Koziel H, Tachado SD (2014). HIV-derived ssRNA binds to TLR8 to induce inflammation-driven macrophage foam cell formation. PLoS ONE.

[67] Krug A, French AR, Barchet W, Fischer JA, Dzionek A, Pingel JT, Orihuela MM, Akira S, Yokoyama WM, Colonna M (2004). TLR9-dependent recognition of MCMV by IPC and DC generates coordinated cytokine responses that activate antiviral NK cell function. Immunity.

[68] Zolini GP, Lima GK, Lucinda N, Silva MA, Dias MF, Pessoa NL, Coura BP, Cartelle CT, Arantes RM, Kroon EG, Campos MA (2014). Defense against HSV-1 in a murine model is mediated by iNOS and orchestrated by the activation of TLR2 and TLR9 in trigeminal ganglia. J Neuroinflammation.

[69] Parroche P, Lauw FN, Goutagny N, Latz E, Monks BG, Visintin A, Halmen KA, Lamphier M, Olivier M, Bartholomeu DC (2007). Malaria hemozoin is immunologically inert but radically enhances innate responses by presenting malaria DNA to Toll-like receptor 9. Proc Natl Acad Sci U S A.

[70] Santamaria MH, Perez Caballero E, Corral RS (2016). Unmethylated CpG motifs in Toxoplasma gondii DNA induce TLR9- and IFN-beta-dependent expression of alpha-defensin-5 in intestinal epithelial cells. Parasitology.

[71] Pohar J, Yamamoto C, Fukui R, Cajnko MM, Miyake K, Jerala R, Bencina M (2017). Selectivity of human TLR9 for double cpg motifs and implications for the recognition of genomic DNA. J Immunol.

[72] Bradshaw MJ, Venkatesan A (2016). Herpes simplex virus-1 encephalitis in adults: pathophysiology, diagnosis, and management. Neurotherapeutics.

[73] Mader J, Gallo A, Schommartz T, Handke W, Nagel CH, Gunther P, Brune W, Reich K (2016). Calcium spirulan derived from Spirulina platensis inhibits herpes simplex virus 1 attachment to human keratinocytes and protects against herpes labialis. J Allergy Clin Immunol.

[74] Koyanagi N, Imai T, Shindo K, Sato A, Fujii W, Ichinohe T, Takemura N, Kakuta S, Uematsu S, Kiyono H (2017). Herpes simplex virus-1 evasion of CD8+ T cell accumulation contributes to viral encephalitis. J Clin Invest.

[75] Majer A, Caligiuri KA, Gale KK, Niu Y, Phillipson CS, Booth TF, Booth SA (2017). Induction of multiple miR-200/182 members in the brains of mice are associated with acute herpes simplex virus 1 encephalitis. PLoS ONE.

[76] Suazo PA, Tognarelli EI, Kalergis AM, Gonzalez PA (2015). Herpes simplex virus 2 infection: molecular association with HIV and novel microbicides to prevent disease. Med Microbiol Immunol.

[77] Hensel MT, Marshall JD, Dorwart MR, Heeke DS, Rao E, Tummala P, Yu L, Cohen GH, Eisenberg RJ, Sloan DD: Prophylactic herpes simplex virus 2 (HSV-2) vaccines adjuvanted with stable emulsion and toll-like receptor 9 agonist induce a robust HSV-2-specific cell-mediated immune response, protect against symptomatic disease, and reduce the latent viral reservoir**.** J Virol 2017, **91**. 10.1128/JVI.02257-16 PMC5391472 28228587

[78] Betta M, Laurino M, Pugliese A, Guzzetta G, Landi A, Manfredi P (2016). Perspectives on optimal control of varicella and herpes zoster by mass routine varicella vaccination. Proc Biol Sci.

[79] Nagel MA, Jones D, Wyborny A (2017). Varicella zoster virus vasculopathy: the expanding clinical spectrum and pathogenesis. J Neuroimmunol.

[80] Dunmire SK, Hogquist KA, Balfour HH (2015). Infectious mononucleosis. Curr Top Microbiol Immunol.

[81] Singavi AK, Harrington AM, Fenske TS (2015). Post-transplant lymphoproliferative disorders. Cancer Treat Res.

[82] Zhang J, Jia L, Tsang CM, Tsao SW (2017). EBV infection and glucose metabolism in nasopharyngeal carcinoma. Adv Exp Med Biol.

[83] Abdelrahim LM, Peh SC, Kallarakkal TG (2018). Epstein-Barr virus infection in B-cell Non-Hodgkin's Lymphomas of the oral and maxillofacial region: is there any evidence?. Malays J Pathol.

[84] Meunier YA (2005). Infectious mononucleosis-like syndrome and gastrointestinal disorders in acute acquired cytomegalovirus infection. Singapore Med J.

[85] Yonezawa A, Onaka T, Imada K (2009). Cytomegalovirus-associated infectious mononucleosis-like syndrome accompanied by transient monoclonal expansion of CD8+ T-cells. Rinsho Ketsueki.

[86] Leach CT (2000). Human herpesvirus-6 and -7 infections in children: agents of roseola and other syndromes. Curr Opin Pediatr.

[87] Razonable RR, Paya CV (2002). The impact of human herpesvirus-6 and -7 infection on the outcome of liver transplantation. Liver Transpl.

[88] Buyse S, Roque-Afonso AM, Vaghefi P, Gigou M, Dussaix E, Duclos-Vallee JC, Samuel D, Guettier C (2013). Acute hepatitis with periportal confluent necrosis associated with human herpesvirus 6 infection in liver transplant patients. Am J Clin Pathol.

[89] Ogata M, Oshima K, Ikebe T, Takano K, Kanamori H, Kondo T, Ueda Y, Mori T, Hashimoto H, Ogawa H (2017). Clinical characteristics and outcome of human herpesvirus-6 encephalitis after allogeneic hematopoietic stem cell transplantation. Bone Marrow Transplant.

[90] Cesarman E, Knowles DM (1997). Kaposi's sarcoma-associated herpesvirus: a lymphotropic human herpesvirus associated with Kaposi's sarcoma, primary effusion lymphoma, and multicentric Castleman's disease. Semin Diagn Pathol.

[91] Said J (1997). Kaposi's sarcoma-associated herpesvirus (KSHV): a new viral pathogen associated with Kaposi's sarcoma, primary effusion lymphoma, and multicentric Castleman's disease. West J Med.

[92] van Rhee F, Wong RS, Munshi N, Rossi JF, Ke XY, Fossa A, Simpson D, Capra M, Liu T, Hsieh RK (2014). Siltuximab for multicentric Castleman's disease: a randomised, double-blind, placebo-controlled trial. Lancet Oncol.

[93] Mariggio G, Koch S, Schulz TF: Kaposi sarcoma herpesvirus pathogenesis**.** Philos Trans R Soc Lond B Biol Sci 2017, **372**. 10.1098/rstb.2016.0275 PMC5597742 28893942

[94] Harris KD (2019). Herpes simplex virus keratitis. Home Healthc Now.

[95] Vitale S, Loubatier C, Cannavo I, Giordanengo V (2019). Problematic molecular diagnosis of HSV-1 infection due to a single nucleotide polymorphism in the US7 gene. J Clin Virol.

[96] Sun L, Li Q (2012). The miRNAs of herpes simplex virus (HSV). Virol Sin.

[97] Peri P, Mattila RK, Kantola H, Broberg E, Karttunen HS, Waris M, Vuorinen T, Hukkanen V (2008). Herpes simplex virus type 1 Us3 gene deletion influences toll-like receptor responses in cultured monocytic cells. Virol J.

[98] van Lint AL, Murawski MR, Goodbody RE, Severa M, Fitzgerald KA, Finberg RW, Knipe DM, Kurt-Jones EA (2010). Herpes simplex virus immediate-early ICP0 protein inhibits Toll-like receptor 2-dependent inflammatory responses and NF-kappaB signaling. J Virol.

[99] Cermelli C, Orsi CF, Ardizzoni A, Lugli E, Cenacchi V, Cossarizza A, Blasi E (2008). Herpes simplex virus type 1 dysregulates anti-fungal defenses preventing monocyte activation and downregulating toll-like receptor-2. Microbiol Immunol.

[100] Jaggi U, Wang S, Tormanen K, Matundan H, Ljubimov AV, Ghiasi H (2018). Role of herpes simplex virus type 1 (HSV-1) glycoprotein K (gK) pathogenic CD8(+) T cells in exacerbation of eye disease. Front Immunol.

[101] Lu X, Huang C, Zhang Y, Lin Y, Wang X, Li Q, Liu S, Tang J, Zhou L (2017). The Us2 gene product of herpes simplex virus 2 modulates NF-kappaB activation by targeting TAK1. Sci Rep.

[102] Guo YJ, Luo T, Wu F, Mei YW, Peng J, Liu H, Li HR, Zhang SL, Dong JH, Fang Y, Zhao L (2015). Involvement of TLR2 and TLR9 in the anti-inflammatory effects of chlorogenic acid in HSV-1-infected microglia. Life Sci.

[103] Wang X, Li Y, Liu S, Yu X, Li L, Shi C, He W, Li J, Xu L, Hu Z (2014). Direct activation of RIP3/MLKL-dependent necrosis by herpes simplex virus 1 (HSV-1) protein ICP6 triggers host antiviral defense. Proc Natl Acad Sci U S A.

[104] Wang JP, Bowen GN, Zhou S, Cerny A, Zacharia A, Knipe DM, Finberg RW, Kurt-Jones EA (2012). Role of specific innate immune responses in herpes simplex virus infection of the central nervous system. J Virol.

[105] Kurt-Jones EA, Chan M, Zhou S, Wang J, Reed G, Bronson R, Arnold MM, Knipe DM, Finberg RW (2004). Herpes simplex virus 1 interaction with Toll-like receptor 2 contributes to lethal encephalitis. Proc Natl Acad Sci U S A.

[106] Sorensen LN, Reinert LS, Malmgaard L, Bartholdy C, Thomsen AR, Paludan SR (2008). TLR2 and TLR9 synergistically control herpes simplex virus infection in the brain. J Immunol.

[107] Leoni V, Gianni T, Salvioli S, Campadelli-Fiume G (2012). Herpes simplex virus glycoproteins gH/gL and gB bind Toll-like receptor 2, and soluble gH/gL is sufficient to activate NF-kappaB. J Virol.

[108] Jin MS, Kim SE, Heo JY, Lee ME, Kim HM, Paik SG, Lee H, Lee JO (2007). Crystal structure of the TLR1-TLR2 heterodimer induced by binding of a tri-acylated lipopeptide. Cell.

[109] Liu H, Chen K, Feng W, Wu X, Li H (2013). TLR4-MyD88/Mal-NF-kB axis is involved in infection of HSV-2 in human cervical epithelial cells. PLoS ONE.

[110] Villalba M, Hott M, Martin C, Aguila B, Valdivia S, Quezada C, Zambrano A, Concha MI, Otth C (2012). Herpes simplex virus type 1 induces simultaneous activation of Toll-like receptors 2 and 4 and expression of the endogenous ligand serum amyloid A in astrocytes. Med Microbiol Immunol.

[111] Brun P, Scarpa M, Marchiori C, Conti J, Kotsafti A, Porzionato A, De Caro R, Scarpa M, Calistri A, Castagliuolo I (2018). Herpes simplex virus type 1 engages toll like receptor 2 to Recruit macrophages during infection of enteric neurons. Front Microbiol.

[112] Strickson S, Emmerich CH, Goh ETH, Zhang J, Kelsall IR, Macartney T, Hastie CJ, Knebel A, Peggie M, Marchesi F (2017). Roles of the TRAF6 and Pellino E3 ligases in MyD88 and RANKL signaling. Proc Natl Acad Sci U S A.

[113] Moon G, Kim J, Min Y, Wi SM, Shim JH, Chun E, Lee KY (2015). Phosphoinositide-dependent kinase-1 inhibits TRAF6 ubiquitination by interrupting the formation of TAK1-TAB2 complex in TLR4 signaling. Cell Signal.

[114] Vollmer S, Strickson S, Zhang T, Gray N, Lee KL, Rao VR, Cohen P (2017). The mechanism of activation of IRAK1 and IRAK4 by interleukin-1 and Toll-like receptor agonists. Biochem J.

[115] Lin SC, Lo YC, Wu H (2010). Helical assembly in the MyD88-IRAK4-IRAK2 complex in TLR/IL-1R signalling. Nature.

[116] Besse A, Lamothe B, Campos AD, Webster WK, Maddineni U, Lin SC, Wu H, Darnay BG (2007). TAK1-dependent signaling requires functional interaction with TAB2/TAB3. J Biol Chem.

[117] Broglie P, Matsumoto K, Akira S, Brautigan DL, Ninomiya-Tsuji J (2010). Transforming growth factor beta-activated kinase 1 (TAK1) kinase adaptor, TAK1-binding protein 2, plays dual roles in TAK1 signaling by recruiting both an activator and an inhibitor of TAK1 kinase in tumor necrosis factor signaling pathway. J Biol Chem.

[118] Sun L, Deng L, Ea CK, Xia ZP, Chen ZJ (2004). The TRAF6 ubiquitin ligase and TAK1 kinase mediate IKK activation by BCL10 and MALT1 in T lymphocytes. Mol Cell.

[119] Kim ML, Jeong HG, Kasper CA, Arrieumerlou C (2010). IKKalpha contributes to canonical NF-kappaB activation downstream of Nod1-mediated peptidoglycan recognition. PLoS ONE.

[120] Mansur DS, Kroon EG, Nogueira ML, Arantes RM, Rodrigues SC, Akira S, Gazzinelli RT, Campos MA (2005). Lethal encephalitis in myeloid differentiation factor 88-deficient mice infected with herpes simplex virus 1. Am J Pathol.

[121] Cai M, Li M, Wang K, Wang S, Lu Q, Yan J, Mossman KL, Lin R, Zheng C (2013). The herpes simplex virus 1-encoded envelope glycoprotein B activates NF-kappaB through the Toll-like receptor 2 and MyD88/TRAF6-dependent signaling pathway. PLoS ONE.

[122] Gianni T, Leoni V, Campadelli-Fiume G (2013). Type I interferon and NF-kappaB activation elicited by herpes simplex virus gH/gL via alphavbeta3 integrin in epithelial and neuronal cell lines. J Virol.

[123] Kim WJ, Choi JW, Jang WJ, Kang YS, Lee CW, Synytsya A, Park YI (2017). Low-molecular weight mannogalactofucans prevent herpes simplex virus type 1 infection via activation of Toll-like receptor 2. Int J Biol Macromol.

[124] Vageli DP, Exarchou A, Zafiriou E, Doukas PG, Doukas S, Roussaki-Schulze A (2015). Effect of TNF-alpha inhibitors on transcriptional levels of pro-inflammatory interleukin-33 and Toll-like receptors-2 and -9 in psoriatic plaques. Exp Ther Med.

[125] Cai MS, Li ML, Zheng CF (2012). Herpesviral infection and Toll-like receptor 2. Protein Cell.

[126] Aravalli RN, Hu S, Rowen TN, Palmquist JM, Lokensgard JR (2005). Cutting edge: TLR2-mediated proinflammatory cytokine and chemokine production by microglial cells in response to herpes simplex virus. J Immunol.

[127] Lv X, Wang H, Su A, Xu S, Chu Y (2018). Herpes simplex virus type 2 infection triggers AP-1 transcription activity through TLR4 signaling in genital epithelial cells. Virol J.

[128] Ahmad R, El Bassam S, Cordeiro P, Menezes J (2008). Requirement of TLR2-mediated signaling for the induction of IL-15 gene expression in human monocytic cells by HSV-1. Blood.

[129] Kurt-Jones EA, Belko J, Yu C, Newburger PE, Wang J, Chan M, Knipe DM, Finberg RW (2005). The role of toll-like receptors in herpes simplex infection in neonates. J Infect Dis.

[130] Schachtele SJ, Hu S, Little MR, Lokensgard JR (2010). Herpes simplex virus induces neural oxidative damage via microglial cell Toll-like receptor-2. J Neuroinflammation.

[131] Reinert LS, Harder L, Holm CK, Iversen MB, Horan KA, Dagnaes-Hansen F, Ulhoi BP, Holm TH, Mogensen TH, Owens T (2012). TLR3 deficiency renders astrocytes permissive to herpes simplex virus infection and facilitates establishment of CNS infection in mice. J Clin Invest.

[132] Willmann O, Ahmad-Nejad P, Neumaier M, Hennerici MG, Fatar M (2010). Toll-like receptor 3 immune deficiency may be causative for HSV-2-associated mollaret meningitis. Eur Neurol.

[133] Tabeta K, Hoebe K, Janssen EM, Du X, Georgel P, Crozat K, Mudd S, Mann N, Sovath S, Goode J (2006). The Unc93b1 mutation 3d disrupts exogenous antigen presentation and signaling via Toll-like receptors 3, 7 and 9. Nat Immunol.

[134] Brinkmann MM, Spooner E, Hoebe K, Beutler B, Ploegh HL, Kim YM (2007). The interaction between the ER membrane protein UNC93B and TLR3, 7, and 9 is crucial for TLR signaling. J Cell Biol.

[135] Kim YM, Brinkmann MM, Paquet ME, Ploegh HL (2008). UNC93B1 delivers nucleotide-sensing toll-like receptors to endolysosomes. Nature.

[136] Pelka K, Bertheloot D, Reimer E, Phulphagar K, Schmidt SV, Christ A, Stahl R, Watson N, Miyake K, Hacohen N (2018). The chaperone UNC93B1 regulates toll-like receptor stability independently of endosomal TLR transport. Immunity.

[137] Vercammen E, Staal J, Beyaert R (2008). Sensing of viral infection and activation of innate immunity by toll-like receptor 3. Clin Microbiol Rev.

[138] Suh HS, Brosnan CF, Lee SC (2009). Toll-like receptors in CNS viral infections. Curr Top Microbiol Immunol.

[139] Mielcarska MB, Bossowska-Nowicka M, Toka FN (2018). Functional failure of TLR3 and its signaling components contribute to herpes simplex encephalitis. J Neuroimmunol.

[140] Weber F, Wagner V, Rasmussen SB, Hartmann R, Paludan SR (2006). Double-stranded RNA is produced by positive-strand RNA viruses and DNA viruses but not in detectable amounts by negative-strand RNA viruses. J Virol.

[141] Wall AA, Luo L, Hung Y, Tong SJ, Condon ND, Blumenthal A, Sweet MJ, Stow JL (2017). Small GTPase Rab8a-recruited Phosphatidylinositol 3-Kinase gamma regulates signaling and cytokine outputs from endosomal toll-like receptors. J Biol Chem.

[142] Sun H, Zhuang G, Chai L, Wang Z, Johnson D, Ma Y, Chen YH (2012). TIPE2 controls innate immunity to RNA by targeting the phosphatidylinositol 3-kinase-Rac pathway. J Immunol.

[143] Yamashita M, Chattopadhyay S, Fensterl V, Saikia P, Wetzel JL, Sen GC (2012). Epidermal growth factor receptor is essential for Toll-like receptor 3 signaling. Sci Signal.

[144] Donepudi M, Resh MD (2008). c-Src trafficking and co-localization with the EGF receptor promotes EGF ligand-independent EGF receptor activation and signaling. Cell Signal.

[145] Koff JL, Shao MX, Ueki IF, Nadel JA (2008). Multiple TLRs activate EGFR via a signaling cascade to produce innate immune responses in airway epithelium. Am J Physiol Lung Cell Mol Physiol.

[146] Johnsen IB, Nguyen TT, Ringdal M, Tryggestad AM, Bakke O, Lien E, Espevik T, Anthonsen MW (2006). Toll-like receptor 3 associates with c-Src tyrosine kinase on endosomes to initiate antiviral signaling. EMBO J.

[147] Naito E, Dewa K, Yamanouchi H, Mitani K, Kominami R (1989). DNA fingerprinting by means of a nonradioactive probe of sulfonated DNA. Nihon Hoigaku Zasshi.

[148] Oshiumi H, Matsumoto M, Funami K, Akazawa T, Seya T (2003). TICAM-1, an adaptor molecule that participates in Toll-like receptor 3-mediated interferon-beta induction. Nat Immunol.

[149] Yamamoto M, Sato S, Hemmi H, Hoshino K, Kaisho T, Sanjo H, Takeuchi O, Sugiyama M, Okabe M, Takeda K, Akira S (2003). Role of adaptor TRIF in the MyD88-independent toll-like receptor signaling pathway. Science.

[150] Joung SM, Park ZY, Rani S, Takeuchi O, Akira S, Lee JY (2011). Akt contributes to activation of the TRIF-dependent signaling pathways of TLRs by interacting with TANK-binding kinase 1. J Immunol.

[151] Han KJ, Su X, Xu LG, Bin LH, Zhang J, Shu HB (2004). Mechanisms of the TRIF-induced interferon-stimulated response element and NF-kappaB activation and apoptosis pathways. J Biol Chem.

[152] Ruckdeschel K, Pfaffinger G, Haase R, Sing A, Weighardt H, Hacker G, Holzmann B, Heesemann J (2004). Signaling of apoptosis through TLRs critically involves toll/IL-1 receptor domain-containing adapter inducing IFN-beta, but not MyD88, in bacteria-infected murine macrophages. J Immunol.

[153] Ueda S, Mineta T, Nakahara Y, Okamoto H, Shiraishi T, Tabuchi K (2004). Induction of the DNA repair gene O6-methylguanine-DNA methyltransferase by dexamethasone in glioblastomas. J Neurosurg.

[154] Sasai M, Oshiumi H, Matsumoto M, Inoue N, Fujita F, Nakanishi M, Seya T (2005). Cutting edge: NF-kappaB-activating kinase-associated protein 1 participates in TLR3/Toll-IL-1 homology domain-containing adapter molecule-1-mediated IFN regulatory factor 3 activation. J Immunol.

[155] Hacker H, Redecke V, Blagoev B, Kratchmarova I, Hsu LC, Wang GG, Kamps MP, Raz E, Wagner H, Hacker G (2006). Specificity in Toll-like receptor signalling through distinct effector functions of TRAF3 and TRAF6. Nature.

[156] Oganesyan G, Saha SK, Guo B, He JQ, Shahangian A, Zarnegar B, Perry A, Cheng G (2006). Critical role of TRAF3 in the Toll-like receptor-dependent and -independent antiviral response. Nature.

[157] Fitzgerald KA, McWhirter SM, Faia KL, Rowe DC, Latz E, Golenbock DT, Coyle AJ, Liao SM, Maniatis T (2003). IKKepsilon and TBK1 are essential components of the IRF3 signaling pathway. Nat Immunol.

[158] Doyle S, Vaidya S, O'Connell R, Dadgostar H, Dempsey P, Wu T, Rao G, Sun R, Haberland M, Modlin R, Cheng G (2002). IRF3 mediates a TLR3/TLR4-specific antiviral gene program. Immunity.

[159] Liu Z, Guan Y, Sun X, Shi L, Liang R, Lv X, Xin W (2013). HSV-1 activates NF-kappaB in mouse astrocytes and increases TNF-alpha and IL-6 expression via Toll-like receptor 3. Neurol Res.

[160] Jiang Z, Zamanian-Daryoush M, Nie H, Silva AM, Williams BR, Li X (2003). Poly(I-C)-induced Toll-like receptor 3 (TLR3)-mediated activation of NFkappa B and MAP kinase is through an interleukin-1 receptor-associated kinase (IRAK)-independent pathway employing the signaling components TLR3-TRAF6-TAK1-TAB2-PKR. J Biol Chem.

[161] Shim JH, Xiao C, Paschal AE, Bailey ST, Rao P, Hayden MS, Lee KY, Bussey C, Steckel M, Tanaka N (2005). TAK1, but not TAB1 or TAB2, plays an essential role in multiple signaling pathways in vivo. Genes Dev.

[162] Meylan E, Burns K, Hofmann K, Blancheteau V, Martinon F, Kelliher M, Tschopp J (2004). RIP1 is an essential mediator of Toll-like receptor 3-induced NF-kappa B activation. Nat Immunol.

[163] Israel A (2010). The IKK complex, a central regulator of NF-kappaB activation. Cold Spring Harb Perspect Biol.

[164] Ma Y, He B (2014). Recognition of herpes simplex viruses: toll-like receptors and beyond. J Mol Biol.

[165] Sato S, Sugiyama M, Yamamoto M, Watanabe Y, Kawai T, Takeda K, Akira S (2003). Toll/IL-1 receptor domain-containing adaptor inducing IFN-beta (TRIF) associates with TNF receptor-associated factor 6 and TANK-binding kinase 1, and activates two distinct transcription factors, NF-kappa B and IFN-regulatory factor-3, in the Toll-like receptor signaling. J Immunol.

[166] Gohda J, Matsumura T, Inoue J (2004). Cutting edge: TNFR-associated factor (TRAF) 6 is essential for MyD88-dependent pathway but not toll/IL-1 receptor domain-containing adaptor-inducing IFN-beta (TRIF)-dependent pathway in TLR signaling. J Immunol.

[167] Hochrein H, Schlatter B, O'Keeffe M, Wagner C, Schmitz F, Schiemann M, Bauer S, Suter M, Wagner H (2004). Herpes simplex virus type-1 induces IFN-alpha production via Toll-like receptor 9-dependent and -independent pathways. Proc Natl Acad Sci U S A.

[168] Lund J, Sato A, Akira S, Medzhitov R, Iwasaki A (2003). Toll-like receptor 9-mediated recognition of Herpes simplex virus-2 by plasmacytoid dendritic cells. J Exp Med.

[169] Wu CC, Lee J, Raz E, Corr M, Carson DA (2004). Necessity of oligonucleotide aggregation for toll-like receptor 9 activation. J Biol Chem.

[170] Latz E, Schoenemeyer A, Visintin A, Fitzgerald KA, Monks BG, Knetter CF, Lien E, Nilsen NJ, Espevik T, Golenbock DT (2004). TLR9 signals after translocating from the ER to CpG DNA in the lysosome. Nat Immunol.

[171] Zeigerer A, Gilleron J, Bogorad RL, Marsico G, Nonaka H, Seifert S, Epstein-Barash H, Kuchimanchi S, Peng CG, Ruda VM (2012). Rab5 is necessary for the biogenesis of the endolysosomal system in vivo. Nature.

[172] Rink J, Ghigo E, Kalaidzidis Y, Zerial M (2005). Rab conversion as a mechanism of progression from early to late endosomes. Cell.

[173] McDermott H, Kim K (2015). Molecular dynamics at the endocytic portal and regulations of endocytic and recycling traffics. Eur J Cell Biol.

[174] Huotari J, Helenius A (2011). Endosome maturation. EMBO J.

[175] Futter CE, Collinson LM, Backer JM, Hopkins CR (2001). Human VPS34 is required for internal vesicle formation within multivesicular endosomes. J Cell Biol.

[176] Vieira OV, Botelho RJ, Rameh L, Brachmann SM, Matsuo T, Davidson HW, Schreiber A, Backer JM, Cantley LC, Grinstein S (2001). Distinct roles of class I and class III phosphatidylinositol 3-kinases in phagosome formation and maturation. J Cell Biol.

[177] Shuto T, Xu H, Wang B, Han J, Kai H, Gu XX, Murphy TF, Lim DJ, Li JD (2001). Activation of NF-kappa B by nontypeable Hemophilus influenzae is mediated by toll-like receptor 2-TAK1-dependent NIK-IKK alpha /beta-I kappa B alpha and MKK3/6-p38 MAP kinase signaling pathways in epithelial cells. Proc Natl Acad Sci U S A.

[178] Takeshita F, Leifer CA, Gursel I, Ishii KJ, Takeshita S, Gursel M, Klinman DM (2001). Cutting edge: Role of Toll-like receptor 9 in CpG DNA-induced activation of human cells. J Immunol.

[179] Jiang Z, Ninomiya-Tsuji J, Qian Y, Matsumoto K, Li X (2002). Interleukin-1 (IL-1) receptor-associated kinase-dependent IL-1-induced signaling complexes phosphorylate TAK1 and TAB2 at the plasma membrane and activate TAK1 in the cytosol. Mol Cell Biol.

[180] Takeshita F, Gursel I, Ishii KJ, Suzuki K, Gursel M, Klinman DM (2004). Signal transduction pathways mediated by the interaction of CpG DNA with Toll-like receptor 9. Semin Immunol.

[181] Gomes MT, Campos PC, Pereira Gde S, Bartholomeu DC, Splitter G, Oliveira SC (2016). TLR9 is required for MAPK/NF-kappaB activation but does not cooperate with TLR2 or TLR6 to induce host resistance to Brucella abortus. J Leukoc Biol.

[182] Ramakrishnan P, Wang W, Wallach D (2004). Receptor-specific signaling for both the alternative and the canonical NF-kappaB activation pathways by NF-kappaB-inducing kinase. Immunity.

[183] Yamaoka S, Courtois G, Bessia C, Whiteside ST, Weil R, Agou F, Kirk HE, Kay RJ, Israel A (1998). Complementation cloning of NEMO, a component of the IkappaB kinase complex essential for NF-kappaB activation. Cell.

[184] Hacker H, Mischak H, Hacker G, Eser S, Prenzel N, Ullrich A, Wagner H (1999). Cell type-specific activation of mitogen-activated protein kinases by CpG-DNA controls interleukin-12 release from antigen-presenting cells. EMBO J.

[185] Hartmann G, Krieg AM (2000). Mechanism and function of a newly identified CpG DNA motif in human primary B cells. J Immunol.

[186] Ruan M, Thorn K, Liu S, Li S, Yang W, Zhang C, Zhang C (2014). The secretion of IL-6 by CpG-ODN-treated cancer cells promotes T-cell immune responses partly through the TLR-9/AP-1 pathway in oral squamous cell carcinoma. Int J Oncol.

[187] Mueller NH, Gilden DH, Cohrs RJ, Mahalingam R, Nagel MA (2008). Varicella zoster virus infection: clinical features, molecular pathogenesis of disease, and latency. Neurol Clin.

[188] Campbell TM, McSharry BP, Steain M, Slobedman B, Abendroth A (2015). Varicella-zoster virus and herpes simplex virus 1 differentially modulate NKG2D ligand expression during productive infection. J Virol.

[189] Wang JP, Kurt-Jones EA, Shin OS, Manchak MD, Levin MJ, Finberg RW (2005). Varicella-zoster virus activates inflammatory cytokines in human monocytes and macrophages via Toll-like receptor 2. J Virol.

[190] Black AP, Jones L, Malavige GN, Ogg GS (2009). Immune evasion during varicella zoster virus infection of keratinocytes. Clin Exp Dermatol.

[191] Yu HR, Huang HC, Kuo HC, Sheen JM, Ou CY, Hsu TY, Yang KD (2011). IFN-alpha production by human mononuclear cells infected with varicella-zoster virus through TLR9-dependent and -independent pathways. Cell Mol Immunol.

[192] Malmgaard L, Melchjorsen J, Bowie AG, Mogensen SC, Paludan SR (2004). Viral activation of macrophages through TLR-dependent and -independent pathways. J Immunol.

[193] Sironi M, Peri AM, Cagliani R, Forni D, Riva S, Biasin M, Clerici M, Gori A (2017). TLR3 mutations in adult patients with herpes simplex virus and varicella-zoster virus encephalitis. J Infect Dis.

[194] Albanese M, Tagawa T, Buschle A, Hammerschmidt W: MicroRNAs of epstein-barr virus control innate and adaptive antiviral immunity. J Virol 2017, **91**. 10.1128/JVI.01667-16 PMC5533892 28592533

[195] van Gent M, Braem SG, de Jong A, Delagic N, Peeters JG, Boer IG, Moynagh PN, Kremmer E, Wiertz EJ, Ovaa H (2014). Epstein-barr virus large tegument protein BPLF1 contributes to innate immune evasion through interference with toll-like receptor signaling. PLoS Pathog.

[196] van Gent M, Gram AM, Boer IGJ, Geerdink RJ, Lindenbergh MFS, Lebbink RJ, Wiertz EJ, Ressing ME (2015). Silencing the shutoff protein of Epstein-Barr virus in productively infected B cells points to (innate) targets for immune evasion. J Gen Virol.

[197] van Gent M, Griffin BD, Berkhoff EG, van Leeuwen D, Boer IG, Buisson M, Hartgers FC, Burmeister WP, Wiertz EJ, Ressing ME (2011). EBV lytic-phase protein BGLF5 contributes to TLR9 downregulation during productive infection. J Immunol.

[198] Gaudreault E, Fiola S, Olivier M, Gosselin J (2007). Epstein-Barr virus induces MCP-1 secretion by human monocytes via TLR2. J Virol.

[199] Ntoufa S, Vilia MG, Stamatopoulos K, Ghia P, Muzio M (2016). Toll-like receptors signaling: A complex network for NF-kappaB activation in B-cell lymphoid malignancies. Semin Cancer Biol.

[200] Li Z, Duan Y, Cheng S, Chen Y, Hu Y, Zhang L, He J, Liao Q, Yang L, Sun LQ (2015). EBV-encoded RNA via TLR3 induces inflammation in nasopharyngeal carcinoma. Oncotarget.

[201] Iwakiri D (2014). Epstein-barr virus-encoded RNAs: key molecules in viral pathogenesis. Cancers (Basel).

[202] Martin HJ, Lee JM, Walls D, Hayward SD (2007). Manipulation of the toll-like receptor 7 signaling pathway by epstein-barr virus. J Virol.

[203] Schoenemeyer A, Barnes BJ, Mancl ME, Latz E, Goutagny N, Pitha PM, Fitzgerald KA, Golenbock DT (2005). The interferon regulatory factor, IRF5, is a central mediator of toll-like receptor 7 signaling. J Biol Chem.

[204] Feederle R, Kost M, Baumann M, Janz A, Drouet E, Hammerschmidt W, Delecluse HJ (2000). The epstein-barr virus lytic program is controlled by the co-operative functions of two transactivators. EMBO J.

[205] Ladell K, Dorner M, Zauner L, Berger C, Zucol F, Bernasconi M, Niggli FK, Speck RF, Nadal D (2007). Immune activation suppresses initiation of lytic Epstein-Barr virus infection. Cell Microbiol.

[206] Traggiai E, Becker S, Subbarao K, Kolesnikova L, Uematsu Y, Gismondo MR, Murphy BR, Rappuoli R, Lanzavecchia A (2004). An efficient method to make human monoclonal antibodies from memory B cells: potent neutralization of SARS coronavirus. Nat Med.

[207] Fiola S, Gosselin D, Takada K, Gosselin J (2010). TLR9 contributes to the recognition of EBV by primary monocytes and plasmacytoid dendritic cells. J Immunol.

[208] Salloum N, Hussein HM, Jammaz R, Jiche S, Uthman IW, Abdelnoor AM, Rahal EA (2018). Epstein-Barr virus DNA modulates regulatory T-cell programming in addition to enhancing interleukin-17A production via Toll-like receptor 9. PLoS ONE.

[209] Dell'Oste V, Biolatti M, Galitska G, Griffante G, Gugliesi F, Pasquero S, Zingoni A, Cerboni C, De Andrea M (2020). Tuning the orchestra: HCMV vs innate immunity. Front Microbiol.

[210] Choi HJ, Park A, Kang S, Lee E, Lee TA, Ra EA, Lee J, Lee S, Park B (2018). Human cytomegalovirus-encoded US9 targets MAVS and STING signaling to evade type I interferon immune responses. Nat Commun.

[211] Britt W (2008). Manifestations of human cytomegalovirus infection: proposed mechanisms of acute and chronic disease. Curr Top Microbiol Immunol.

[212] Fu YZ, Su S, Gao YQ, Wang PP, Huang ZF, Hu MM, Luo WW, Li S, Luo MH, Wang YY, Shu HB (2017). Human cytomegalovirus tegument protein UL82 Inhibits STING-mediated signaling to evade antiviral immunity. Cell Host Microbe.

[213] Park A, Ra EA, Lee TA, Choi HJ, Lee E, Kang S, Seo JY, Lee S, Park B (2019). HCMV-encoded US7 and US8 act as antagonists of innate immunity by distinctively targeting TLR-signaling pathways. Nat Commun.

[214] Skert C, Fogli M, Garaffa E, Perucca S, Fiorentini S, Cancelli V, Turra A, Ribolla R, Fili C, Malagola M (2014). A specific Toll-like receptor profile on T lymphocytes and values of monocytes correlate with bacterial, fungal, and cytomegalovirus infections in the early period of allogeneic stem cell transplantation. Transpl Infect Dis.

[215] Smith PD, Shimamura M, Musgrove LC, Dennis EA, Bimczok D, Novak L, Ballestas M, Fenton A, Dandekar S, Britt WJ, Smythies LE (2014). Cytomegalovirus enhances macrophage TLR expression and MyD88-mediated signal transduction to potentiate inducible inflammatory responses. J Immunol.

[216] Becker M, Lemmermann NA, Ebert S, Baars P, Renzaho A, Podlech J, Stassen M, Reddehase MJ (2015). Mast cells as rapid innate sensors of cytomegalovirus by TLR3/TRIF signaling-dependent and -independent mechanisms. Cell Mol Immunol.

[217] Landais I, Pelton C, Streblow D, DeFilippis V, McWeeney S, Nelson JA (2015). Human cytomegalovirus miR-UL112-3p targets TLR2 and modulates the TLR2/IRAK1/NFkappaB signaling pathway. PLoS Pathog.

[218] Compton T, Kurt-Jones EA, Boehme KW, Belko J, Latz E, Golenbock DT, Finberg RW (2003). Human cytomegalovirus activates inflammatory cytokine responses via CD14 and Toll-like receptor 2. J Virol.

[219] Boehme KW, Guerrero M, Compton T (2006). Human cytomegalovirus envelope glycoproteins B and H are necessary for TLR2 activation in permissive cells. J Immunol.

[220] Brown RA, Gralewski JH, Razonable RR (2009). The R753Q polymorphism abrogates toll-like receptor 2 signaling in response to human cytomegalovirus. Clin Infect Dis.

[221] Borden EC, Sen GC, Uze G, Silverman RH, Ransohoff RM, Foster GR, Stark GR (2007). Interferons at age 50: past, current and future impact on biomedicine. Nat Rev Drug Discov.

[222] Zhang L, Yu J, Liu Z (2020). MicroRNAs expressed by human cytomegalovirus. Virol J.

[223] Gatot JS, Gioia R, Chau TL, Patrascu F, Warnier M, Close P, Chapelle JP, Muraille E, Brown K, Siebenlist U (2007). Lipopolysaccharide-mediated interferon regulatory factor activation involves TBK1-IKKepsilon-dependent Lys(63)-linked polyubiquitination and phosphorylation of TANK/I-TRAF. J Biol Chem.

[224] Cohen L, Henzel WJ, Baeuerle PA (1998). IKAP is a scaffold protein of the IkappaB kinase complex. Nature.

[225] Watters TM, Kenny EF, O'Neill LA (2007). Structure, function and regulation of the Toll/IL-1 receptor adaptor proteins. Immunol Cell Biol.

[226] Kawagoe T, Sato S, Matsushita K, Kato H, Matsui K, Kumagai Y, Saitoh T, Kawai T, Takeuchi O, Akira S (2008). Sequential control of Toll-like receptor-dependent responses by IRAK1 and IRAK2. Nat Immunol.

[227] Loiarro M, Gallo G, Fanto N, De Santis R, Carminati P, Ruggiero V, Sette C (2009). Identification of critical residues of the MyD88 death domain involved in the recruitment of downstream kinases. J Biol Chem.

[228] Ngo VN, Young RM, Schmitz R, Jhavar S, Xiao W, Lim KH, Kohlhammer H, Xu W, Yang Y, Zhao H (2011). Oncogenically active MYD88 mutations in human lymphoma. Nature.

[229] Hemmi H, Takeuchi O, Kawai T, Kaisho T, Sato S, Sanjo H, Matsumoto M, Hoshino K, Wagner H, Takeda K, Akira S (2000). A Toll-like receptor recognizes bacterial DNA. Nature.

[230] Krieg AM (2002). CpG motifs in bacterial DNA and their immune effects. Annu Rev Immunol.

[231] De Bolle L, Naesens L, De Clercq E (2005). Update on human herpesvirus 6 biology, clinical features, and therapy. Clin Microbiol Rev.

[232] Zerr DM (2006). Human herpesvirus 6: a clinical update. Herpes.

[233] Furukawa M, Yasukawa M, Yakushijin Y, Fujita S (1994). Distinct effects of human herpesvirus 6 and human herpesvirus 7 on surface molecule expression and function of CD4+ T cells. J Immunol.

[234] Hasegawa A, Yasukawa M, Sakai I, Fujita S (2001). Transcriptional down-regulation of CXC chemokine receptor 4 induced by impaired association of transcription regulator YY1 with c-Myc in human herpesvirus 6-infected cells. J Immunol.

[235] Murakami Y, Tanimoto K, Fujiwara H, An J, Suemori K, Ochi T, Hasegawa H, Yasukawa M (2010). Human herpesvirus 6 infection impairs Toll-like receptor signaling. Virol J.

[236] Zandi E, Rothwarf DM, Delhase M, Hayakawa M, Karin M (1997). The IkappaB kinase complex (IKK) contains two kinase subunits, IKKalpha and IKKbeta, necessary for IkappaB phosphorylation and NF-kappaB activation. Cell.

[237] El-Ela MA, Shaarawy E, El-Komy M, Fawzy M, Hay RA, Hegazy R, Sharobim A, Moustafa N, Rashed L, Sayed Amr KS (2016). Is there a link between human herpesvirus infection and toll-like receptors in the pathogenesis of pityriasis rosea? A case-control study. Acta Dermatovenerol Croat.

[238] Prantsidis A, Rigopoulos D, Papatheodorou G, Menounos P, Gregoriou S, Alexiou-Mousatou I, Katsambas A (2009). Detection of human herpesvirus 8 in the skin of patients with pityriasis rosea. Acta Derm Venereol.

[239] Ueda K (2018). KSHV genome replication and maintenance in latency. Adv Exp Med Biol.

[240] Jacobs SR, Gregory SM, West JA, Wollish AC, Bennett CL, Blackbourn DJ, Heise MT, Damania B (2013). The viral interferon regulatory factors of kaposi's sarcoma-associated herpesvirus differ in their inhibition of interferon activation mediated by toll-like receptor 3. J Virol.

[241] Meyer F, Ehlers E, Steadman A, Waterbury T, Cao M, Zhang L (2013). TLR-TRIF pathway enhances the expression of KSHV replication and transcription activator. J Biol Chem.

[242] Lingel A, Ehlers E, Wang Q, Cao M, Wood C, Lin R, Zhang L (2016). Kaposi's sarcoma-associated herpesvirus reduces cellular myeloid differentiation primary-response gene 88 (MyD88) expression via modulation of Its RNA. J Virol.

[243] West J, Damania B (2008). Upregulation of the TLR3 pathway by Kaposi's sarcoma-associated herpesvirus during primary infection. J Virol.

[244] West JA, Gregory SM, Sivaraman V, Su L, Damania B (2011). Activation of plasmacytoid dendritic cells by Kaposi's sarcoma-associated herpesvirus. J Virol.

[245] Gruffaz M, Vasan K, Tan B (2017). TLR4-mediated inflammation promotes KSHV-induced cellular transformation and tumorigenesis by activating the STAT3 pathway. Cancer Res.

[246] Lagos D, Vart RJ, Gratrix F, Westrop SJ, Emuss V, Wong PP, Robey R, Imami N, Bower M, Gotch F, Boshoff C (2008). Toll-like receptor 4 mediates innate immunity to Kaposi sarcoma herpesvirus. Cell Host Microbe.

[247] Abend JR, Ramalingam D, Kieffer-Kwon P, Uldrick TS, Yarchoan R, Ziegelbauer JM (2012). Kaposi's sarcoma-associated herpesvirus microRNAs target IRAK1 and MYD88, two components of the toll-like receptor/interleukin-1R signaling cascade, to reduce inflammatory-cytokine expression. J Virol.

[248] Bussey KA, Reimer E, Todt H, Denker B, Gallo A, Konrad A, Ottinger M, Adler H, Sturzl M, Brune W, Brinkmann MM (2014). The gammaherpesviruses Kaposi's sarcoma-associated herpesvirus and murine gammaherpesvirus 68 modulate the Toll-like receptor-induced proinflammatory cytokine response. J Virol.

[249] Lore K, Betts MR, Brenchley JM, Kuruppu J, Khojasteh S, Perfetto S, Roederer M, Seder RA, Koup RA (2003). Toll-like receptor ligands modulate dendritic cells to augment cytomegalovirus- and HIV-1-specific T cell responses. J Immunol.

[250] Lucinda N, Figueiredo MM, Pessoa NL, Santos BS, Lima GK, Freitas AM, Machado AM, Kroon EG, Antonelli LR, Campos MA (2017). Dendritic cells, macrophages, NK and CD8(+) T lymphocytes play pivotal roles in controlling HSV-1 in the trigeminal ganglia by producing IL1-beta, iNOS and granzyme B. Virol J.

[251] Kawai T, Akira S (2007). Antiviral signaling through pattern recognition receptors. J Biochem.

[252] Bernstein DI, Cardin RD, Bravo FJ, Earwood J, Clark JR, Li Y, Mishra P, Li C, Nayak BP, Miller AT (2014). Topical SMIP-7.7, a toll-like receptor 7 agonist, protects against genital herpes simplex virus type-2 disease in the guinea pig model of genital herpes. Antivir Chem Chemother.

[253] Dendouga N, Fochesato M, Lockman L, Mossman S, Giannini SL (2012). Cell-mediated immune responses to a varicella-zoster virus glycoprotein E vaccine using both a TLR agonist and QS21 in mice. Vaccine.

[254] Jo BR, Yu JM, Jang S, Ahn JW, Kim HS, Seoung EA, Park HY, Jin DH, Joo SS (2020). Cloning, expression, and purification of a pathogenesis-related protein from oenanthe javanica and its biological properties. Biol Pharm Bull.

[255] Su AR, Qiu M, Li YL, Xu WT, Song SW, Wang XH, Song HY, Zheng N, Wu ZW (2017). BX-795 inhibits HSV-1 and HSV-2 replication by blocking the JNK/p38 pathways without interfering with PDK1 activity in host cells. Acta Pharmacol Sin.

