# Correction to: Toll-like receptor-mediated innate immunity against herpesviridae infection: a current perspective on viral infection signaling pathways

**DOI:** 10.1186/s12985-020-01481-0

**Published:** 2020-12-31

**Authors:** Wenjin Zheng, Qing Xu, Yiyuan Zhang, Xiaofei E, Wei Gao, Mogen Zhang, Weijie Zhai, Ronaldjit Singh Rajkumar, Zhijun Liu

**Affiliations:** 1 School of Basic Medical Sciences, Weifang Medical University, Weifang, 261053 China; 2 School of Anesthesiology, Weifang Medical University, Weifang, 261053 China; 3 Department of Microbiology and Physiological Systems, University of Massachusetts Medical School, Worcester, MA 01605 USA; 4 Key Lab for Immunology in Universities of Shandong Province, School of Basic Medical Sciences, Weifang Medical University, Weifang, 261053 China; 5 Department of Medical Microbiology, School of Basic Medical Sciences, Weifang Medical University, Weifang, 261053 China

## Correction to: Virol J (2020) 17:192 https://doi.org/10.1186/s12985-020-01463-2

Following publication of the original article [1], the authors identified an error in the author name of Xiaofei E. The given name and family name were erroneously transposed.

The incorrect author name is: E Xiaofei.

The correct author name is: Xiaofei E.

The author group has been updated above and the original article [1] has been corrected.

## References

[1] Zheng et al. Virol J (2020) 17:192. 10.1186/s12985-020-01463-2

